# Exploring small non-coding RNAs as blood-based biomarkers to predict Alzheimer’s disease

**DOI:** 10.1186/s13578-023-01190-5

**Published:** 2024-01-16

**Authors:** Laia Gutierrez-Tordera, Christopher Papandreou, Nil Novau-Ferré, Pablo García-González, Melina Rojas, Marta Marquié, Luis A. Chapado, Christos Papagiannopoulos, Noèlia Fernàndez-Castillo, Sergi Valero, Jaume Folch, Miren Ettcheto, Antoni Camins, Mercè Boada, Agustín Ruiz, Mònica Bulló

**Affiliations:** 1 Nutrition and Metabolic Health Research Group, Department of Biochemistry and Biotechnology, Rovira i Virgili University (URV), 43201 Reus, Spain; 2 Institute of Health Pere Virgili (IISPV), 43204 Reus, Spain; 3 Center of Environmental, Food and Toxicological Technology-TecnATox, Rovira i Virgili University, 43201 Reus, Spain; 4 ACE Alzheimer Center Barcelona, Universitat Internacional de Catalunya (UIC), 08028 Barcelona, Spain; 5 Biomedical Research Networking Centre in Neurodegenerative Diseases (CIBERNED), Carlos III Health Institute, 28031 Madrid, Spain; 6 Laboratory of Epigenetics of Lipid Metabolism, Instituto Madrileño de Estudios Avanzados (IMDEA)-Alimentación, CEI UAM+CSIC, 28049 Madrid, Spain; 7 Department of Hygiene and Epidemiology, University of Ioannina School of Medicine, 45500 Ioannina, Greece; 8 Department de Genetics, Microbiology and Statistics, Faculty of Biology, Universitat de Barcelona, 08007 Barcelona, Spain; 9 Department of Pharmacology, Toxicology and Therapeutic Chemistry, Faculty of Pharmacy and Food Science, Universitat de Barcelona, 08028 Barcelona, Spain; 10 Institute of Neuroscience, Universitat de Barcelona, 08035 Barcelona, Spain; 11 CIBER Physiology of Obesity and Nutrition (CIBEROBN), Carlos III Health Institute, 28029 Madrid, Spain

**Keywords:** Alzheimer’s disease, Mild cognitive impairment, ATN, Biomarkers, Small non-coding RNA, Gene regulatory networks, Nested case–control study

## Abstract

**Background:**

Alzheimer’s disease (AD) diagnosis relies on clinical symptoms complemented with biological biomarkers, the Amyloid Tau Neurodegeneration (ATN) framework. Small non-coding RNA (sncRNA) in the blood have emerged as potential predictors of AD. We identified sncRNA signatures specific to ATN and AD, and evaluated both their contribution to improving AD conversion prediction beyond ATN alone.

**Methods:**

This nested case–control study was conducted within the ACE cohort and included MCI patients matched by sex. Patients free of type 2 diabetes underwent cerebrospinal fluid (CSF) and plasma collection and were followed-up for a median of 2.45-years. Plasma sncRNAs were profiled using small RNA-sequencing. Conditional logistic and Cox regression analyses with elastic net penalties were performed to identify sncRNA signatures for A+(T|N)+ and AD. Weighted scores were computed using cross-validation, and the association of these scores with AD risk was assessed using multivariable Cox regression models. Gene ontology (GO) and Kyoto encyclopaedia of genes and genomes (KEGG) enrichment analysis of the identified signatures were performed.

**Results:**

The study sample consisted of 192 patients, including 96 A+(T|N)+ and 96 A-T-N- patients. We constructed a classification model based on a 6-miRNAs signature for ATN. The model could classify MCI patients into A-T-N- and A+(T|N)+ groups with an area under the curve of 0.7335 (95% CI, 0.7327 to 0.7342). However, the addition of the model to conventional risk factors did not improve the prediction of AD beyond the conventional model plus ATN status (C-statistic: 0.805 [95% CI, 0.758 to 0.852] compared to 0.829 [95% CI, 0.786, 0.872]). The AD-related 15-sncRNAs signature exhibited better predictive performance than the conventional model plus ATN status (C-statistic: 0.849 [95% CI, 0.808 to 0.890]). When ATN was included in this model, the prediction further improved to 0.875 (95% CI, 0.840 to 0.910). The miRNA-target interaction network and functional analysis, including GO and KEGG pathway enrichment analysis, suggested that the miRNAs in both signatures are involved in neuronal pathways associated with AD.

**Conclusions:**

The AD-related sncRNA signature holds promise in predicting AD conversion, providing insights into early AD development and potential targets for prevention.

**Supplementary Information:**

The online version contains supplementary material available at 10.1186/s13578-023-01190-5.

## Background

Dementia is the 7th leading cause of death worldwide, with Alzheimer’s disease (AD) accounting for the 60–70% of cases [1]. Less severe forms of cognitive dysfunction, including mild cognitive impairment (MCI), usually precede the development of dementia. In fact, participants with MCI display three to five times higher risk for dementia progression, mostly Alzheimer, compared to those with normal cognition [2]. Therefore, the identification of blood-based early detection biomarkers of MCI and risk of progression to AD is a challenge for more accurate and personalised medicine.

In 2011, the National Institute on Aging and Alzheimer’s Association agreed on the ATN classification system, based on biological markers of disease rather than on its clinical consequences, in an attempt to better address the complexity of the disease [3]. Several studies have demonstrated that memory declines faster in the A + T + N + and A+(T|N)+ groups compared with the other five ATN profiles [4]. Thus, the ATN system offers an opportunity to improve the prediction of short-term memory decline and AD progression. Added to the clinical MCI diagnosis, the ATN framework has the advantage of discriminating between groups of participants, which represents a promising tool for future diagnosis and therapeutic-targeted treatments. Despite the promise of the ATN classification system for improving the prediction of AD progression, its complexity and discomfort during the lumbar puncture induce limits to its widespread use in clinical settings [5]. Therefore, there is a critical need for the identification of less invasive blood-based biomarkers to improve early detection and prediction of AD progression [6]. Plasma levels of Aβ proteins, p-tau217 or p-tau181 have been identified as significantly higher in participants with early or mild AD, demonstrating high accuracy in distinguishing AD from several other neurodegenerative diseases [7–10]. The performance of plasma P-tau alone accurately predicted AD dementia within 4 years (AUC = 0.83), and this prediction was further improved when combined with APOE genotype and cognitive tests (AUC = 0.91) [11]. Small RNAs, particularly microRNAs (miRNAs) and transfer RNA-derived small RNAs (tRNAs) have also attracted considerable attention as potential biomarkers and therapeutic targets [12, 13]. Combining them with ATN biomarkers may offer an approach to improve the prediction of AD conversion. The utilization of circulating miRNAs as multimarker panels is becoming a less expensive and time-consuming diagnosis tool and offers insights into the underlying molecular mechanisms of diseases. Due to their role as genetic regulators, miRNAs are nowadays considered potential therapeutic tools for restoring cell functions disrupted during disease progression [14–16]. Previous studies have shown that specific sncRNAs have differential expression between AD patients and cognitively healthy participants [17–21], and some of them exhibit good predictive accuracy [22]. Furthermore, differential expression of sncRNAs has been observed between individuals with MCI and healthy participants [19, 23, 24]. However, few studies have investigated sncRNAs beyond miRNA in AD prediction, and none have compared the additive value of ATN-related and AD-related sncRNA signatures. The ATN-related sncRNA signature may reflect specific pathological processes that lead to AD, such as Aβ and P-tau accumulation. Conversely, the AD-related sncRNA signature captures a broader spectrum of molecular changes associated with AD progression. Incorporating both signatures could help to identify individuals at different stages of AD development.

In this study, we aimed to investigate the utility of sncRNA signatures related to AD or ATN classification in improving the prediction of AD conversion compared to ATN alone. We identified an AD-related sncRNA signature capable of predicting AD conversion and compared its predictive performance with that of the ATN-related sncRNA signature.

## Methods

### Study design and population

This study has been conducted within the ACE cohort, which consists of MCI men and women aged > 48 years recruited and assessed between 2006 to 2022 at the Memory Disorders Unit (ACE Alzheimer Center, Spain). Patients were diagnosed with MCI at a case conference attended by neurologists, neuropsychologists, and social workers, and using the Spanish version of the Mini-Mental State Examination (MMSE), the memory part of the Spanish version of the 7 Minute test, the Spanish version of the Neuropsychiatric Inventory Questionnaire (NPI-Q), the Hachinski Ischemia Scale, the Blessed Dementia Scale and the Clinical Dementia Rating (CDR) scale, as well as a comprehensive neuropsychological battery of ACE (N-BACE) [25] Detailed characteristics of the study population can be found elsewhere [26]. From a total of 361 participants with MCI, free of type 2 diabetes, who had ATN classification, available plasma samples and follow-up data, 211 individuals were classified as A+(T|N)+ . For this nested case–control study, we randomly selected 96 A+(T|N)+ and their paired 96 A-T-N- participants matched by sex (Additional file 1: eFigure 1). Lumbar punctures were performed at ACE Alzheimer Center under fasting conditions. Aβ and tau proteins were quantified by either the commercially available enzyme-linked immunosorbent assays (ELISAs) (INNOTEST, Fujirebio Europe) or the chemiluminescence enzyme immunoassay (CLEIA) using the Lumipulse G 600 II automatic platform (Fujirebio Europe). Cut-offs to dichotomize each CSF biomarker into ± were as follows: for ELISA, Aβ1-42 < 676 pg/mL for A, p181-Tau > 58 pg/mL for T and T-Tau > 367 pg/mL for N; for CLEIA, Aβ1-42 < 796 pg/mL for A; p181-tau > 54 pg/mL for T and T-tau > 412 pg/mL for N [27]. Glucose and insulin concentrations were measured in plasma samples (Institute of Health Carlos III, ACE Alzheimer Center collection C.0000299), using standard enzymatic automated methods. Ascertainment of AD was assigned by consensus at a case conference using the same procedures and questionnaires as for MCI, plus the 2011 NIA-AA for Alzheimer’s disease. During a median follow-up time of 2.45 years (IQR = 2.17), 74 incident AD events occurred. Information about demographic characteristics and lifestyle factors (i.e., age, sex, BMI, *APOE* ε4, education, smoking habit, and medication) was collected. The clinical dataset was downloaded on 15 February 2023.

### Data processing

We identified 1981 sncRNAs through small RNA sequencing (Additional file 1: eMethods). After applying data filtering and excluding those transcripts with less than 1000 total reads, a total of 208 sncRNAs remained available for further analysis. One patient was excluded from the control group due to a low number of reads in its sample, resulting in a total of 191 participants (Additional file 1: eFigure 1). Its paired case was also removed from the subsequent matched case–control analyses. Abnormally sncRNA expression values beyond z-score ± 3 were excluded from the analyses [28]. Missing data was imputed by applying the random forest approach using the missForest package (v1.5) [29]. Reads were normalized for sequencing depth, gene length, and RNA composition using the DESeq2 package (v.1.38.2) [30] and z-score was calculated to generate comparable effect sizes between sncRNAs. Due to the number of sncRNAs, P values were adjusted for multiple testing using the false discovery rate approach (Benjamini–Hochberg method) [31]. Significance was set at Padj < 0.05.

### Statistical analyses

Descriptive data is shown as median and interquartile range for quantitative variables, and percentages for categorical variables. The distribution of variables was assessed using the Kolmogorov–Smirnov test. Differences between matched case–control groups were assessed using Wilcoxon’s Signed Rank Test and McNemar’s test for continuous and categorical variables, respectively. Group differences between independent variables were examined using the Mann–Whitney test and the chi-square test, respectively.

#### Univariate analyses

The differential expression and association of sncRNAs with ATN status and AD were examined in univariate analyses. The DESeq2 package was used to identify differentially expressed sncRNAs between A+(T|N)+ versus A-T-N- and between AD-converters and non-converters. Unadjusted conditional logistic regression analysis was performed to examine the association between sncRNAs and A+(T|N)+ . Adjusted conditional logistic regression analysis was also performed to examine the independent associations between sncRNAs and A+(T|N)+ . The model included several covariates such as age, BMI, *APOE* ε4, education, smoking habit, use of anxiolytic, antidepressants or antihypertensive drugs, use of statins or other lipid-lowering medication, and MMSE score. Odds ratios (ORs) and their 95% CIs were calculated considering A-T-N- as the reference category. Sensitivity analyses using unconditional logistic regressions were also performed to examine whether these associations were modified by the matching factor; sex. For these analyses, we used the aforementioned adjusted models additionally adjusted for sex. Cox regression analysis adjusting for the previous covariates plus ATN at baseline was used to identify sncRNAs related to the risk of progression from MCI to AD. Hazard ratios (HRs) and their 95% CIs were estimated.

#### Multivariate analyses

Given the high dimensionality of the data and the multicollinearity among the sncRNA (Additional file 1: eFigure 2), we also performed multivariate analyses. In order to identify sncRNA signatures for A+(T|N)+ and AD, we used a regularized regression approach using the “glmnet” package (v4.1.6) [32]. The estimated required sample size to find associations between the ATN-related signature and A+(T|N)+ was 192 (OR 1.56, 80% statistical power, P value = 0.05). The estimated required number of AD events to find associations between the ATN-related signature or the AD-related signature and AD was 73 (HR 2.05, 80% statistical power, P value = 0.05). We employed elastic net regression modeling that has the ability to reduce complexity of high dimensional data and handle multicollinearity by performing variable selection and regularization simultaneously. The elastic net method combines the L1 and the L2 penalties of the Lasso and Ridge methods. This method finds the ridge regression coefficients and then conducts the second step by using a Lasso sort of shrinkage of the coefficients, which tends to choose one variable from highly correlated groups and ignore the rest [33]. To identify a sncRNA signature for ATN, we scaled the data and regressed ATN status on the 208 sncRNAs using a conditional logistic regression with elastic net penalty. To confirm our results, a sensitivity analysis using a logistic regression with elastic net penalty was further performed for the 191 participants with available data. Thereafter, to identify a sncRNA signature for AD, we performed a Cox regression analysis with elastic net penalty. To reduce overfitting, the data was divided into training set (90%) and test set (10%). In the training set, we evaluated the alpha parameter from 0.1 to 1 in ~ 0.05 increments (avoiding L2 norm), and the tuning parameter (lambda), using a tenfold cross-validation (CV) approach in a 20-iteration loop. The ATN signature and the AD signature had an alpha value of 0.90 and 0.65, respectively, and a lambda value of 0.058724638 and 0.030235403, respectively. The combination of alpha and lambda was chosen based on a high Area Under the ROC Curve (AUC) and C-statistic in the test set. We applied the selected alpha and lambda values to each elastic net regression for every training of a 100-iteration loop. We built the ATN signature and AD signature only with those sncRNAs that were consistently selected in more than 90 iterations. For each sncRNA selected from the model, we calculated the mean coefficient and the 95% CI. The AUC and the C-statistic were calculated in a 1000-iteration loop to evaluate the performance of the sncRNA profile in assessing ATN and AD incidence, respectively.

Finally, the coefficients obtained from 90 or more iterations in the elastic net were applied to the selected sncRNAs as weights (positive or negative) to estimate the signatures of ATN and AD as the weighted sum. Before Cox regression analyses, the identified signatures (sncRNA score) were transformed by z-score (mean = 0; SD = 1). For our analyses of AD incidence, the time-to-event variable was the interval between the date of enrolment and the date of the AD event. We examined associations of ATN status and the two signatures with AD risk fitting two adjusted Cox regression models (adjusted for age, sex, BMI, *APOE* ε4, smoking habit, education, use of anxiolytic or antidepressants, antihypertensive drugs, statins, and other lipid-lowering medication, and baseline MMSE) to estimate HRs and 95% CIs. To assess the added predictive ability of the derived signatures, we compared through likelihood ratio tests the C-statistics between the first model including the conventional AD risk factors plus ATN and the other models including each signature in addition to these risk factors. All analyses were conducted using R software (v. 4.2.1) (R Foundation for Statistical Computing, Vienna, Austria).

### Functional enrichment analysis

The minimum network with the strongest evidenced target genes of sncRNAs linked to AD signatures was identified through the validated miRTarBase [34] and TarBase [35] databases, incorporated in the miRNet platform [36]. The protein–protein (PPI) network was added to the genes network. To further clarify the potential function annotation and pathway enrichment associated with both signatures’ miRNAs, Gene Ontology (GO) analyses, including biological process (BP), molecular function (MF), and cellular component (CC), and Kyoto Encyclopedia of Genes and Genomes (KEGG) pathways, were performed to figure out the functional roles of these miRNAs in WebGestalt platform [37] (significant as FDR < 0.05). To obtain more robust results, genes in the validated miRTarBase database, plus genes that overlapped in the predicted miRDB and TargetScan databases were used. Only miRNA functional enrichment analysis was performed due to the lack of robust gene databases for non-miRNA sncRNAs. A literature search was carried out using the MEDLINE-PubMed database to extract gene functions related to MCI or AD using an algorithm described in Additional file 1: eFigure 3. The search strategy combined the miRNA’s and/or the gene’s name with terms related to Alzheimer’s disease, cognition and brain.

## Results

### Baseline characteristics of the study participants

Descriptive characteristics are shown in Table 1. A+(T|N)+ participants were more likely to be older, to have a lower BMI, to be carriers of *APOE* ε4, were taking less antidepressant and anxiolytic medication, and had lower baseline scores of MMSE compared to A-T-N- participants. Also, A+(T|N)+ participants had a shorter median follow-up period (1.7 vs. 3.1 years) and a higher conversion to AD (71.6% vs. 5.3%). In Table 2 we show the baseline characteristics of AD converters compared to non-converters. A total of 74 MCI participants were converted to AD during the follow-up period. Additional file 1: eTable 1 show the baseline characteristics of participants in the A+(T|N)+ subgroups, consisting of 65 A+T+N+ and 31 A+T+N- or A+T-N+ participants.

**Table 1:** Characteristics of the study participants according to the ATN status

Variable	A-T-N- participants	A+(T|N)+ participants	P value
n	95	95	
Age (years)	69.2 (61.7, 76.2)	76.6 (72.4, 80.1)	< 0.001***
Women [N (%)]	46 (48.0)	46 (48.0)	1.000
Body mass index (kg/m^2^)	27.2 (24.6, 29.2)	25.5 (23.5, 28.5)	0.036*
*APOE* ε4 carriers [N (%)]	13 (13.7)	58 (61.1)	< 0.001***
Education (years)	8 (6, 11)	8 (6, 11)	0.642
Smoking [N (%)]			
Never	65 (68.4)	67 (70.5)	0.880
Former	18 (18.9)	22 (23.2)	0.584
Current	12 (12.6)	6 (6.3)	0.239
Medication [N (%)]			
Antidepressant and anxiolytic	56 (58.9)	32 (33.7)	< 0.001***
Antihypertensive	33 (34.7)	39 (41.1)	0.480
Statins	24 (25.3)	28 (29.5)	0.617
Other lipid-lowering drugs	6 (6.3)	7 (7.4)	1.000
MMSE at baseline (score)	27 (26, 29)	25 (24, 27)	< 0.001***
A [N (%)]	1 (1.1)	95 (100.0)	< 0.001***
T [N (%)]	0 (0.0)	92 (96.8)	< 0.001***
N [N (%)]	0 (0.0)	67 (70.5)	< 0.001***
Follow-up (years)	3.1 (2.2, 4.0)	1.7 (1.0, 2.8)	< 0.001***
Conversion to AD [N (%)]	5 (5.3)	68 (71.6)	< 0.001***

*MMSE* mini-mental state examination, *AD* Alzheimer’s disease

Continuous data are presented as median (interquartile range), and categorical variables are presented as number (%). The Wilcoxon Signed-Rank test was used for comparison of non-normally distributed continuous variables, and the McNemar’s test was used for comparison of categorical variables. *P value < 0.05, **P value < 0.01, ***P value < 0.001

**Table 2:** Characteristics of the study participants according to conversion from MCI to AD

Variable	AD non-converters	AD converters	P value
n	117	74	
Age (years)	69.8 (62.4, 76.4)	77.7 (72.8, 80.6)	< 0.001***
Women [N (%)]	59 (50.4)	34 (45.9)	0.546
Body mass index (kg/m^2^)	27.0 (24.0, 28.8)	25.5 (23.5, 28.7)	0.140
*APOE* ε4 carriers [N (%)]	26 (22.0)	46 (62.2)	< 0.001***
Education (years)	8 (6, 12)	8 (6, 10)	0.143
Smoking [N (%)]			
Never	85 (72.6)	48 (64.9)	0.254
Former	19 (16.2)	21 (28.4)	0.045*
Current	13 (11.1)	5 (6.8)	0.316
Medication [N (%)]			
Antidepressant and anxiolytic	61 (52.1)	27 (36.5)	0.035*
Antihypertensive	37 (31.6)	35 (47.3)	0.029*
Statins	28 (23.9)	24 (32.4)	0.199
Other lipid-lowering drugs	8 (6.8)	5 (6.8)	0.983
MMSE at baseline (score)	27 (25, 28)	25 (23, 27)	< 0.001***
A [N (%)]	28 (23.7)	69 (93.2)	< 0.001***
T [N (%)]	27 (22.9)	66 (89.2)	< 0.001***
N [N (%)]	18 (15.3)	50 (67.6)	< 0.001***
Follow-up (years)	3.1 (2.2, 3.8)	1.3 (1.0, 2.5)	< 0.001***

*AD* Alzheimer’s disease, *MCI* mild cognitive impairment, *MMSE* mini-mental state examination

Continuous data are presented as median (interquartile range), and categorical variables are presented as number (%). The Mann–Whitney test was used for comparison of non-normally distributed continuous variables, and the X^2^ test was used for comparison of categorical variables. *P value < 0.05, **P value < 0.01, ***P value < 0.001

### Associations of sncRNAs with ATN status

#### Univariate analyses

The DESeq2 method was applied to select the most relevant differentially expressed sncRNAs between A+(T|N)+ and A-T-N- groups (Additional file 1: eTable 2). Thirteen sncRNAs (ten upregulated and three downregulated) with unadjusted P values (P value < 0.05) were selected (Additional file 1: eFigure 4a). However, we did not observe any sncRNAs that were differentially expressed when using adjusted P value, as shown in the volcano plot in Additional file 1: eFigure 4b.

Univariate conditional logistic regression analyses were conducted to explore associations between sncRNAs and A+(T|N)+ (Additional file 1: eTable S3). Four sncRNAs (hsa-miR-27b-5p, hsa-miR-339-5p, hsa-miR-548ag, U4) were positively and two (piR-31924, LSU-rRNA-Hsa) negatively associated with A+(T|N)+ . Nonetheless, after multiple testing correction, none of the sncRNAs remained significant. Analyses adjusting for the matching factor also yielded similar results (Additional file 1: eTable 4).

#### Multivariate analyses

**Fig. 1:**
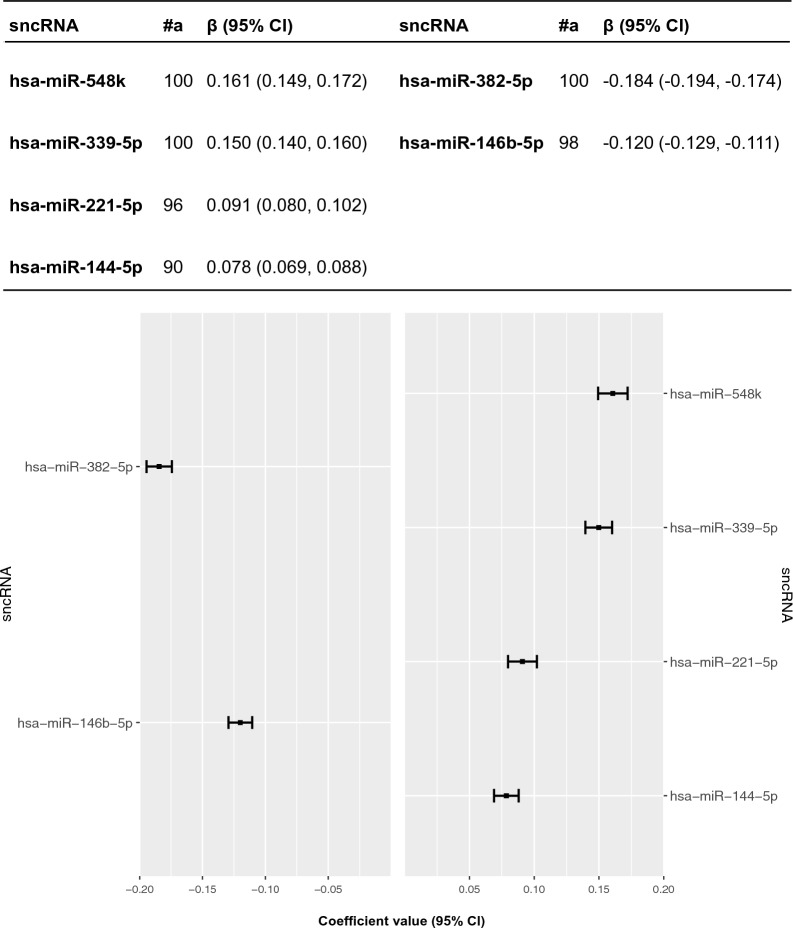
SncRNAs ranked from highest to lowest elastic net regression coefficients for A+(T|N)+ . #a, Occurrence of miRNAs (out of 100) in the elastic net conditional logistic regression, *sncRNA* small non-coding RNA, *hsa* Homo sapiens, *CI* confidence interval

Figure 1 presents the six sncRNAs selected 90–100 times in the elastic net regression analysis for A+(T|N)+ . All of them were miRNAs, namely hsa-miR-548 k, hsa-miR-339-5p, hsa-miR-221-5p and hsa-miR-144-5p with positive coefficients, while hsa-miR-382-5p and hsa-miR-146b-5p had negative coefficients. The AUC of the miRNAs signature for ATN was 0.7335 (95% CI, 0.7327 to 0.7342). In the sensitivity analysis conducted using logistic regression, only four miRNAs (hsa-miR-548 k, hsa-miR-339-5p, hsa-miR-382-5p, hsa-miR-146b-5p) were identified (Additional file 1: eTable 5). These four miRNAs exhibited consistent associations in the same direction as observed in the main analysis. However, the other two miRNAs identified in the conditional logistic regression analysis (hsa-miR-221-5p, hsa-miR-144-5p) were not found to be associated with A+(T|N)+ in the sensitivity analysis.

### Associations of sncRNAs with AD incidence

#### Univariate analyses

We investigated whether sncRNAs could distinguish between participants with MCI who converted to AD and those who did not. The comparison of sncRNA expression levels between 74 AD-converters and 117 non-converters revealed 27 sncRNAs transcripts that were differentially expressed with unadjusted P value < 0.05 (Additional file 1: eTable 6 and Additional file 1: eFigure 5a). However, after controlling for FDR, only two sncRNAs (hsa-miR-151a-5p, hsa-miR-548 k) remained differentially expressed. The upregulated sncRNAs are displayed in the volcano plot (Additional file 1: eFigure 5b).

Among the 11 sncRNAs that exhibited a significant association with AD incidence, only one miRNA (hsa-miR-584-5p) remained significant after adjusting for FDR in the adjusted Cox regression analyses (Additional file 1: eTable 7).

#### Multivariate analyses

**Fig. 2:**
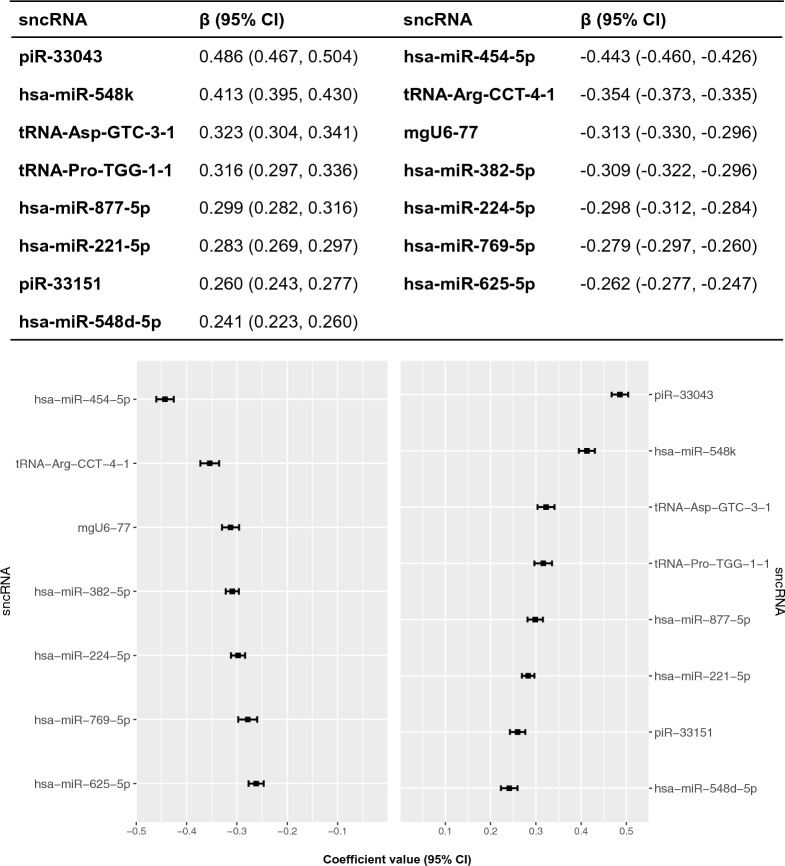
SncRNAs ranked from highest to lowest elastic net regression coefficients for risk of AD. *sncRNA* small non-coding RNA, *piR* piwi RNA, *tRNA* transfer RNA, *hsa* Homo sapiens, *CI* confidence interval

Figure 2 shows the 15 sncRNAs selected in the 10-CV elastic net regression for AD incidence (C-statistic = 0.7948, 95% CI, 0.7942 to 0.7953). Out of the 100 iterations conducted, we observed that none of these 208 sncRNAs were selected fewer than 100 times. Nine of the selected sncRNAs associated with AD risk were miRNAs. Positive associations were observed for four miRNAs (hsa-miR-221-5p, hsa-miR-548d-5p, hsa-miR-548 k, hsa-miR-877-5p), two piRNAs (piR-33043, piR-33151) and two tRNAs (tRNA-Asp-GTC-3-1, tRNA-Pro-TGG-1-1). Five miRNAs (hsa-miR-224-5p, hsa-miR-382-5p, hsa-miR-454-5p, hsa-miR-625-5p, hsa-miR-769-5p), one snoRNA (mgU6-77) and one tRNA (tRNA-Arg-CCT-4-1) were negatively associated with AD.

### Associations of the ATN- and AD-related sncRNA signatures with AD incidence

In the adjusted analyses, we observed a significant association of model 2 (including conventional risk factors and ATN) with AD (Tables 3 and 4). The association between the ATN-related sncRNA signature, including risk factors, and AD was significant, however, this association became insignificant after further adjustment for ATN status (Table 3). In contrast, the association between the AD-related sncRNA signature, including risk factors, and AD remained significant even after additional adjustment for ATN (Table 4). Including the ATN-related sncRNA signature into the conventional model significantly improved the C-statistic for AD (from 0.791 to 0.805, P value = 0.004). On the other hand, adding the ATN-related sncRNA signature to model 2 did not significantly change the prediction precision for AD (from 0.829 to 0.832, P value = 0.292 for the difference between the two C-statistics) (Table 3). The addition of the AD-related sncRNA signature to the conventional model increased the C-statistic for AD from 0.791 to 0.849 (P value < 0.001). Furthermore, when the AD-related sncRNA signature was added to the conventional model plus ATN, the C-statistic further increased from 0.829 to 0.875 (P value < 0.001) (Table 4).

**Table 3:** Associations and performance of the ATN-related sncRNA signature with AD incidence

Analysis model	HR (95% CI)	P value^a^	C-statistic	P value^b^		
				Model 1	Model 2	Model 3
Model 1			0.791 (0.740, 0.842)			
Model 2	3.896 (2.326, 6.524)	< 0.001***	0.829 (0.786, 0.872)	< 0.001***		
Model 3	1.497 (1.141, 1.963)	0.004**	0.805 (0.758, 0.852)	0.003**		
Model 4	1.170 (0.874, 1.567)	0.292	0.832 (0.789, 0.875)	< 0.001***	0.291	< 0.001***

Model 1 included age, sex, BMI, *APOE* ε4, smoking status, education, use of anxiolytic or antidepressants, antihypertensive drugs, statins, and other lipid-lowering medication and MMSE score; Model 2 included ATN status plus the conventional risk factors included in Model 1; Model 3 included the ATN-related sncRNA signature plus the conventional risk factors included in Model 2; Model 4 included the ATN-related sncRNA signature plus the conventional risk factors included in Model 2 and ATN status. Exposure contrast is per z-score increase in the sncRNA signature. *P value < 0.05, **P value < 0.01, ***P value < 0.001

*AD* Alzheimer’s disease, *HR* hazard ratio, *BMI* Body mass index, *MMSE* mini-mental state examination

^a^P value for the hazard ratio

^b^P value for the likelihood-ratio test

**Table 4:** Associations and performance of the AD-related sncRNA signature with AD incidence

Analysis model	HR (95% CI)	P value^a^	C-statistic	P value^b^		
				Model 1	Model 2	Model 3
Model 1			0.791 (0.740, 0.842)			
Model 2	3.896 (2.326, 6.524)	< 0.001***	0.829 (0.786, 0.872)	< 0.001***		
Model 3	3.043 (2.185, 4.239)	< 0.001***	0.849 (0.808, 0.890)	< 0.001***		
Model 4	2.853 (2.020, 4.029)	< 0.001***	0.875 (0.840, 0.910)	< 0.001***	< 0.001***	< 0.001***

Model 1 included age, sex, BMI, *APOE* ε4, smoking status, education, use of anxiolytic or antidepressants, antihypertensive drugs, statins, and other lipid-lowering medication and MMSE score; Model 2 included ATN status plus the conventional risk factors included in Model 1; Model 3 included the AD-related sncRNA signature plus the conventional risk factors included in Model 2; Model 4 included the AD-related sncRNA signature plus the conventional risk factors included in Model 2 and ATN status. Exposure contrast is per z-score increase in the sncRNA signature. *P value < 0.05, **P value < 0.01, ***P value < 0.001

*AD* Alzheimer’s disease, *HR* hazard ratio, *BMI* Body mass index, *MMSE* mini-mental state examination.

^a^P value for the hazard rati

^b^P value for the likelihood-ratio test

### Functional enrichment analysis

**Fig. 3:**
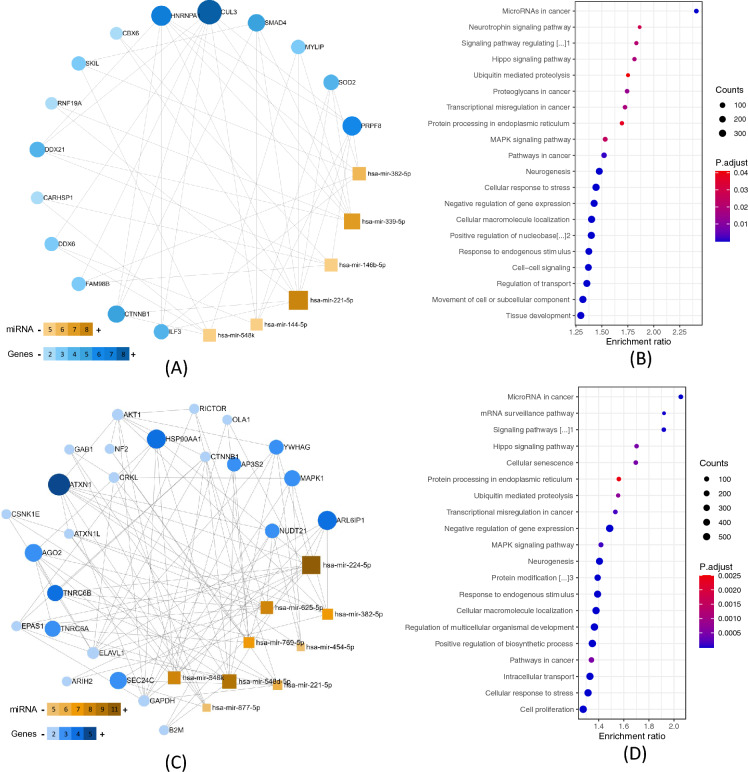
Network construction and functional enrichment analysis of target genes. **A** The network diagram of the ATN-related signature of AD (orange for miRNAs signature, blue for target genes). **B** The significant KEGG pathways and GO functions of target genes for the ATN-related signature of AD (FDR, Padj < 0.05). **C** The network diagram of the AD-related signature (orange for miRNAs signature, blue for target genes). **D** The significant KEGG pathways and GO functions of target genes for the AD-related signature (FDR, Padj < 0.05). Counts represent the number of genes that overlap with the gene set. ^1^Signaling pathways regulating pluripotency of stem cells; ^2^Positive regulation of nucleobase-containing compound metabolic process; ^3^Protein modification by small protein conjugation or removal; *AD* Alzheimer’s disease, *KEGG* Kyoto encyclopaedia of genes and genomes, *GO* Gene ontology

Gene-miRNA interactions, GO functional and KEGG enrichment analyses were performed on ATN-related and AD-related signatures to estimate their molecular function. The gene-miRNA interaction revealed 15 hub genes for the ATN-related sncRNA signature (Fig. 3a) and 25 for the AD-related sncRNA signature (Fig. 3c). All of them were related to neuronal functions (Additional file 1: eTables 8, 9). Their biological processes and pathways were involved in *Hippo* and *MAPK* signalling pathways, neurogenesis and ubiquitin-mediated proteolysis in both signatures, and to other pathways such as the neurotrophin signalling pathway in the ATN-related sncRNA signature (Fig. 3b and Additional file 1: eTable 10) and the mRNA surveillance pathway in the AD-related sncRNA signature (Fig. 3d and Additional file 1: eTable 11). Some of the molecular functions also involved tissue development (Additional file 1: eTable 12) and the cellular component part of a neuron (Additional file 1: eTable 13). These results suggest alterations in the expression of genes related to key metabolic and physiologic processes in neurons of MCI participants with progression to AD, that in turn seems to be regulated by ATN-related sncRNA signature integrated by 6 miRNAs.

## Discussion

The study identified a new signature of 6 miRNAs associated with A+(T|N)+ and with a predictive performance comparable to the ATN classification. Furthermore, the study discovered another signature consisting of 15 sncRNAs that was found to be associated with AD. When this AD-related signature was incorporated into a conventional risk prediction model, with or without ATN biomarkers, it resulted in significant improvements in predicting AD risk. These findings suggest that the AD-related signature may serve as a valuable and novel candidate marker for AD.

Biomarkers play an important role in AD diagnosis and prognosis. Because of its minimal invasiveness and relatively low cost, the use of peripheral blood for AD diagnosis has gained attention [38, 39]. While CSF biomarkers have been studied in relation to AD diagnosis and staging [40], no study has analysed plasma sncRNAs in relation to A+(T|N)+ and AD incidence. We propose a 6-sncRNA signature as an alternative to CSF biomarker analysis for discriminating A+(T|N)+ participants. The AUC analysis suggests that this signature could identify participants with worse AD prognoses using less invasive procedures. Compared to a conventional model with ATN classification, the addition of the ATN-related sncRNA signature showed slightly lower performance. On the other hand, the associations of the ATN-related sncRNA signature with AD were attenuated and became non-significant after adjusting for ATN, suggesting that the miRNAs in this signature do not biologically underlie this association.

Previous studies have reported decreased hsa-miR-339-5p in the brain tissue of AD patients and AD mice models [41]. This miRNA is known to down-regulate *BACE1* expression in human brain cells [41]. In our study, hsa-miR-339-5p was positively associated with A+(T|N)+ signature and MCI to AD conversion. Furthermore, Spearman’s correlation analysis revealed a significant negative correlation between hsa-miR-339-5p and Aβ_42_ (*r* = − 0.192, P value = 0.008). These findings suggest that increased hsa-miR-339-5p expression could be interpreted as a mechanism to counteract Aβ formation, before clinical symptoms develop.

On the contrary, ADAM10 plays a role in the non-amyloidogenic pathway by cleaving the Aβ protein precursor. A study demonstrated that hsa-miR-221 overexpression in SH-SY5Y cells reduced ADAM10 levels [42]. Interestingly, we observed an upregulation of hsa-miR-221-5p in both the A+(T|N)+ signature and the conversion from MCI to AD, potentially serving as a mechanism to decrease ADAM10 and favour AD pathology. Similarly, miR-144 upregulation in vitro reduced ADAM10 [43] and hsa-miR-144-3p was increased in the hippocampi and prefrontal cortex of APP/PS1 mice, leading to cholinergic neuron degeneration, one of the key hallmarks of AD [44]. Hsa-miR-144-5p has been found either upregulated or downregulated in AD patients depending on the analytical method used [45]. Considering our finding of hsa-miR-144-5p targeting *CUL3*, its downregulation leads to increased NFR2 activity [46] a potential novel target for AD treatment due to its antioxidant capacity and role in memory and synaptic plasticity protection [47]. Therefore, we propose that the positive association between the A+(T|N)+ signature and AD progression would involve the modulation of CUL3 and NFR2.

This study is the first to reveal an increased expression of hsa-miR-548 k associated with cognitive impairment.

Hsa-miR-548 k targets genes that are involved in Aβ uptake and clearance by microglia, such as IDOL [48, 49], or regulation of inflammatory processes, such as ADAMTS1 [50, 51], both linked to neurodegenerative diseases. Therefore, we could speculate that a potential upregulation of this miRNA might play a role in the development of AD. The potential implications of hsa-miR-548 k expression in the mechanisms underlying AD development deserve further investigation.

In a case–control study, hsa-miR-382 was positively associated with MCI and effectively discriminated participants with MCI from cognitively healthy participants (AUC = 0.92) [23]. In our study, hsa-miR-382-5p expression levels were inversely associated with A+(T|N)+ and AD risk. This finding is noteworthy considering that hsa-miR-382-5p targets the *EEF1A1* gene, which plays a role in long-term synaptic plasticity and memory consolidation. Hence, the downregulation of hsa-miR-382-5p could be a counteracting mechanism aimed at protecting neurons and maintaining synaptic plasticity to preserve cognitive function [52].

Higher circulating levels of hsa-miR-146a has been consistently reported in MCI participants compared to those with AD [53–55]. In a mouse model of AD, the overexpression of hsa-miR-146a alleviated Aβ deposition and rescued cognitive impairment [56, 57]. In our study we found that hsa-miR-146b-5p was negatively associated with A+(T|N)+ signature, suggesting that the expression of this miRNA might decrease before a diagnosis of AD. In addition, the overexpression of hsa-miR-146b-5p could be a potential strategy to alleviate the pathological changes associated with AD.

Previous studies have demonstrated a relationship between the ratio of plasma levels of Aβ42/40 and cerebral amyloidosis [58], with an AUC of 0.86 [59]. Notably, in our study the addition of the AD-related sncRNA signature to ATN biomarkers modestly improved the risk prediction (C-statistic = 0.875), suggesting that sncRNAs have the potential to assist in the identification of individuals at high risk of AD.

Our results also suggest that the AD-related sncRNA signature captures a broader spectrum of molecular changes associated with AD progression, beyond the accumulation of Aβ and P-tau. The miRNAs (hsa-miR-548 k, hsa-miR-221-5p and hsa-miR-382-5p) found to overlap between the ATN-related and the AD-related sncRNA signatures may reflect early changes in AD pathology related to Aβ and P-tau. In contrast, the presence of other sncRNAs within the AD-related signature suggests that they may capture additional biological events that occur later during AD development.

In addition to the overlapping miRNAs, our study also identified several other miRNAs that showed significant associations with AD. Specifically, we observed decreased expression of hsa-miR-224-5p, hsa-miR-625-5p, hsa-miR-769-5p and hsa-miR-454-5p, while increased expression of hsa-miR-548d-5p and hsa-miR-877-5p. Of particular interest, hsa-miR-224-5p showed the highest number of interactions with target genes. Previous research has shown decreased expression of hsa-miR-224-5p in Aβ_1-42_ microvascular endothelial cells of the blood–brain barrier (BBB), and its upregulation after the memantine treatment, contributing to the amelioration of the BBB permeability in the AD microenvironment [60]. Thus, the decreased expression of hsa-miR-224-5p observed in our study may indicate BBB damage. In line with our results, hsa-miR-877-5p was reported to be upregulated in the synaptosomes of AD post-mortem mice brains [61].

In human post-mortem brains of patients with MCI and AD, hsa-miR-454-3p was found to be upregulated [62]. In our study, hsa-miR-454-5p was inversely associated with the risk of AD conversion, suggesting that during the early stages of AD, hsa-miR-454-5p may counteract AD physiopathology. In addition, Hsa-miR-454-5p hsa-miR-769-5p, and hsa-miR-382-5p, which were also inversely associated with risk of AD conversion, target *MAPK1.* The *MAPK1* signaling pathway has been implicated in the development of AD [63].

To our knowledge, this is the first time that hsa-miR-548 k, hsa-miR-548d-5p, hsa-miR-625-5p and hsa-miR-769-5p are associated with AD. Hsa-miR-548d-5p downregulates *PPARγ* in hBMSCs cells [64] and *PPARγ* agonist treatment reduced amyloid plaque burden, inflammation, and improved cognition in animal models of AD [65]. The upregulation of hsa-miR-548d-5p we found, suggest this miRNA downregulates *PPARγ* and leads to AD pathophysiology.

The upregulation of hsa-miR-625-5p and hsa-miR-769-5p have been related with apoptosis in several types of cancer. Whether they could play a similar role in AD deserves further investigation.

In addition to miRNAs, our AD-related signature included other sncRNAs such as piRNAs, tRNAs, and snoRNAs, which have been less studied in AD. In fast-progressing amyotrophic lateral sclerosis (ALS) piR-33151 was upregulated compared to slow-progressing ALS [66]. Similarly, we found a positive association of both piR-33151and piR-33043 with AD, suggesting that these piRNAs may increase their expression in neurodegenerative disorders due to stress responses. Furthermore, tRNAs are abundant in neural tissue, although they are largely understudied in the context of neurological diseases [67]. Similarly, although mgU6-77 has not been described in relation to AD, it might be involved in methylation processes occurring in this disease [68].

Our study has several strengths such as the prospective design and the use of regularized regression methods to select biologically relevant sncRNAs. Also, the untargeted approach employed in our study ensures comprehensive coverage of the whole blood sncRNA transcriptome, facilitating the discovery of novel sncRNAs associated with AD. The study has also some limitations. First, due to the cross-sectional analysis of the relationships between sncRNAs and ATN, causality cannot be inferred. Second, the A+(T|N)+ profiles may not encompass all individuals at AD risk, suggesting that other types of pathology might be involved in the development of the disease. Third, considering that 15% of MCI participants develop AD after 2 years [69] and about 33% within 5 years [70], incorporating a longer follow-up period would strengthen the predictive value of the sncRNA signatures. Also, performing RNA-Seq analysis of the gene transcriptome would provide additional robustness to our results and better capture the potential interactions between the 15 identified miRNAs signature in the upstream regulator of gene expression. Finally, more confirmatory work is needed before the sncRNA signatures can be applied to the community population. The identified signatures should be validated in independent larger populations and using qPCR. The sncRNAs’ targets should also be validated in vivo and in vitro using AD models. These approaches would help strengthen our findings.

## Conclusions

In summary, our study provides compelling evidence for the incremental predictive value of sncRNA profiling in AD risk prediction. While the ATN-related signature was based on the classical hallmarks of AD (amyloid, tau, and neurodegeneration), it omitted other possible mechanisms leading to AD. Thus, the AD-related signature captured a more extensive spectrum of molecular changes, and not only those related to the accumulation of Aβ or hyperphosphorylated tau. The inclusion of 15 sncRNAs in a prediction model that comprises conventional risk factors and ATN biomarkers led to a notable improvement in AD risk prediction.

Furthermore, the identification of the AD-related sncRNA signature offers an opportunity to deepen our understanding of the molecular mechanisms underlying AD development and paves the way for the discovery of non-invasive blood-based biomarkers that could significantly enhance prevention and treatment efforts.

### Supplementary Information

**Additional file 1: eFigure 1.** Flowchart of patient inclusion. **eMethods.** Sequencing and processing of sncRNA and data processing. **eFigure 2.** Heatmap of sncRNAs correlations. Spearman’s rank correlation coefficient is shown for each correlation. Only significant correlations are shown (P value < .05). **eFigure 3.** A literature search was carried out using the MEDLINE-PubMed database from inception through 10 April 2023. For the database searches, terms related to “miRNA” or “gene” were combined with terms related to Alzheimer’s disease, cognition and brain. In each run, the miRNA’s and the gene name were changed. **eTable 1.** Characteristics of the A+(T|N)+ study participants according to their A, T and N profile. **eTable 2.** Upregulated and downregulated Differentially Expressed sncRNAs comparing A+(T|N)+ with A-T-N-. **eFigure 4.** Volcano plot of upregulated and downregulated Differentially Expressed sncRNAs comparing A+(T|N)+ with A-T-N-. (A) sncRNAs with P value < .05, (B) sncRNAs with Padj < .05 after FDR. **eTable 3.** Conditional logistic regression analysis examining the individual associations between sncRNAs expression and A+(T|N)+. **eTable 4.** Sensitivity analysis using logistic regression analysis to examine the individual associations between sncRNAs expression and A+(T|N)+. **eTable 5.** Sensitivity analysis of sncRNAs associated with A+(T|N)+ in a logistic regression analysis. SncRNA are ranked from the highest to the lowest elastic net positive and negative regression coefficients for A+(T|N)+. **eTable 6.** Upregulated and downregulated Differentially Expressed sncRNAs between AD-converters and AD non-converters. **eFigure 5.** Volcano plot of upregulated and downregulated Differentially Expressed sncRNAs comparing AD-converters and AD non-converters. (A) sncRNAs with P value < .05, (B) sncRNAs with Padj < .05 after FDR. **eTable 7.** Cox regression analysis examining the individual associations between sncRNAs expression and risk of progression from MCI to AD. **eTable 8.** Genes regulated by the ATN-related sncRNA signature. **eTable 9.** Genes regulated by the AD-related sncRNA signature. **eTable 10.** Significantly enriched KEGG pathways for the ATN-related sncRNA signature. **eTable 11.** Significantly enriched KEGG pathways for the AD-related sncRNA signature. **eTable 12.** Significantly enriched GO terms in the ATN-related sncRNA signature. **eTable 13.** Significantly enriched GO terms in the AD-related sncRNA signature.

## Data Availability

Further data will be provided under request to the corresponding author (MBB).

## Abbreviations

AD: Alzheimer’s disease
MCI: Mild cognitive impairment
sncRNA: Small non-coding RNA
MMSE: Mini-Mental State Examination
BMI: Body Mass Index
KEGG: Kyoto Encyclopaedia of Genes and Genomes
