# Supplementary material for: Exploring small non-coding RNAs as blood-based biomarkers to predict Alzheimer’s disease
Source: Cell Biosci. 2024 Jan 16;14:8. doi: 10.1186/s13578-023-01190-5 (PMC10790437; doi:10.1186/s13578-023-01190-5)
Supplement: Supplementary file 1 — Additional file 1: eFigure 1. Flowchart of patient inclusion. eMethods. Sequencing and processing of sncRNA and data processing. eFigure 2. Heatmap of sncRNAs correlations. Spearman’s rank correlation coefficient is shown for each correlation. Only significant correlations are shown (P value < .05). eFigure 3. A literature search was carried out using the MEDLINE-PubMed database from inception through 10 April 2023. For the database searches, terms related to “miRNA” or “gene” were combined with terms related to Alzheimer’s disease, cognition and brain. In each run, the miRNA’s and the gene name were changed. eTable 1. Characteristics of the A+(T|N)+ study participants according to their A, T and N profile. eTable 2. Upregulated and downregulated Differentially Expressed sncRNAs comparing A+(T|N)+ with A-T-N-. eFigure 4. Volcano plot of upregulated and downregulated Differentially Expressed sncRNAs comparing A+(T|N)+ with A-T-N-. (A) sncRNAs with P value < .05, (B) sncRNAs with Padj < .05 after FDR. eTable 3. Conditional logistic regression analysis examining the individual associations between sncRNAs expression and A+(T|N)+. eTable 4. Sensitivity analysis using logistic regression analysis to examine the individual associations between sncRNAs expression and A+(T|N)+. eTable 5. Sensitivity analysis of sncRNAs associated with A+(T|N)+ in a logistic regression analysis. SncRNA are ranked from the highest to the lowest elastic net positive and negative regression coefficients for A+(T|N)+. eTable 6. Upregulated and downregulated Differentially Expressed sncRNAs between AD-converters and AD non-converters. eFigure 5. Volcano plot of upregulated and downregulated Differentially Expressed sncRNAs comparing AD-converters and AD non-converters. (A) sncRNAs with P value < .05, (B) sncRNAs with Padj < .05 after FDR. eTable 7. Cox regression analysis examining the individual associations between sncRNAs expression and risk of progression from MCI to AD. eTable 8. Genes regul [file 13578_2023_1190_MOESM1_ESM.pdf]

ADDITIONAL FILES

eFigure 1. Flowchart of patient inclusion.

eMethods. Sequencing and processing of sncRNA and data processing.

eFigure 2. Heatmap of sncRNAs correlations. Spearman's rank correlation coefficient is shown for each correlation. Only significant correlations are shown (P value < .05).

eFigure 3. A literature search was carried out using the MEDLINE-PubMed database from inception through 10 April 2023. For the database searches, terms related to "miRNA" or "gene" were combined with terms related to Alzheimer's disease, cognition and brain. In each run, the miRNA's and the gene name were changed.

eTable 1. Characteristics of the A+(T|N)+ study participants according to their A, T and N profile.

eTable 2. Upregulated and downregulated Differentially Expressed sncRNAs comparing A+(T|N)+ with A-T-N-.

eFigure 4. Volcano plot of upregulated and downregulated Differentially Expressed sncRNAs comparing A+(T|N)+ with A-T-N-. (A) sncRNAs with P value < .05, (B) sncRNAs with Padj < .05 after FDR.

eTable 3. Conditional logistic regression analysis examining the individual associations between sncRNAs expression and A+(T|N)+.

eTable 4. Sensitivity analysis using logistic regression analysis to examine the individual associations between sncRNAs expression and A+(T|N)+.

eTable 5. Sensitivity analysis of sncRNAs associated with A+(T|N)+ in a logistic regression analysis. SncRNA are ranked from the highest to the lowest elastic net positive and negative regression coefficients for A+(T|N)+.

eTable 6. Upregulated and downregulated Differentially Expressed sncRNAs between AD-converters and AD non-converters.

eFigure 5. Volcano plot of upregulated and downregulated Differentially Expressed sncRNAs comparing AD-converters and AD non-converters. (A) sncRNAs with P value < .05, (B) sncRNAs with Padj < .05 after FDR.

eTable 7. Cox regression analysis examining the individual associations between sncRNAs expression and risk of progression from MCI to AD.

eTable 8. Genes regulated by the ATN-related sncRNA signature.

eTable 9. Genes regulated by the AD-related sncRNA signature.

eTable 10. Significantly enriched KEGG pathways for the ATN-related sncRNA signature.

eTable 11. Significantly enriched KEGG pathways for the AD-related sncRNA signature.

eTable 12. Significantly enriched GO terms in the ATN-related sncRNA signature.

eTable 13. Significantly enriched GO terms in the AD-related sncRNA signature.

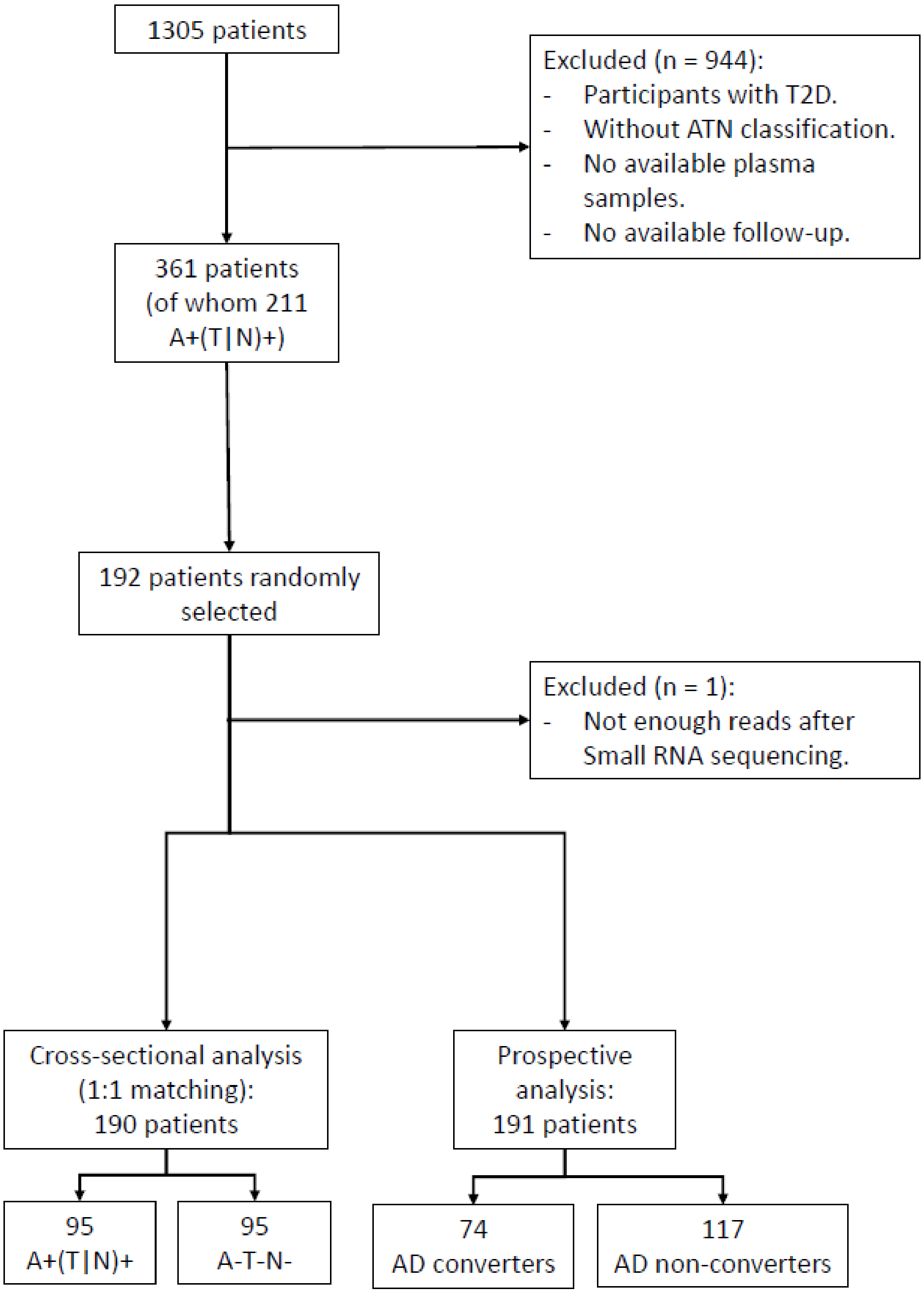

eFigure 1 | Flowchart of patient inclusion.

Abbreviations: T2D, type 2 diabetes; AD, Alzheimer's disease.

eMethods | Sequencing and processing of sncRNA.

Total RNA was isolated from 400 µL of plasma using the Plasma/Serum RNA Purification Midi Kit (Norgen Biotek, Canada) and quantified with the Qubit RNA HS Assay Kit (Life Technologies, USA) on a Qubit fluorometer (Life Technologies, USA). Since the concentration of RNA was below the lower standard, the entire volume was concentrated in vacuo using the Savant SPD2010 Speedvac Concentrator (ThermoScientific, USA) and resuspended in 5 µL of DNase/RNase-free water. SncRNAs were analyzed using Illumina TruSeq SncRNA Library Prep Kit (Illumina, USA), following the manufacturer's instructions. Briefly, RA5 and RA3 RNA oligonucleotides were ligated to 5' and 3' ends of RNA, respectively. Adapter-ligated RNA was reverse-transcribed using an RT Primer and the resulting cDNA was amplified in a 15-cycle PCR that used RP1 and indexed RP1 primers. A gel purification step was used to isolate PCR products of 145-160 bp through a 5% Mini-Protean TBE Gel (Biorad, USA). The integrity of the generated sncRNA sequencing library was confirmed using High Sensitivity D1000 Screen Tape Assay on 4200 TapeStation System (Agilent, USA), and it was quantified with the Qubit 1X dsDNA HS Assay Kit (Life Technologies, USA). The sncRNA expression profiling was generated from the NextSeq 2000 Sequencing System (Illumina, USA). Leftover RNA and cDNA were stored at -80ºC.

The Illumina DRAGEN Bio-IT Platform was used for the generation of raw, demultiplexed sequencing data. Quality control was performed using the FastQC (v0.11.9) software. Reads with a quality score of 20 or higher were filtered using Trim Galore tool (v0.6.7), which implements the Cutadapt tool for adapter trimming.

The mapping and quantification of sncRNAs were done in two different protocols: miRNA and other sncRNAs. For the identification of miRNAs, reads were mapped to the human genome(1) and quantified using mature and hairpin miRNA database references(2) using the miRDeep2 tool (v2.0.1.2). Duplicates were removed. For the identification of other sncRNAs, a BLAST (v.2.5.0) against the miRNA database was performed(2), kept the non-miRNA reads and a new BLAST against the sncRNA database(3) was performed.

eFigure 2 | Heatmap of sncRNAs correlations. Spearman's rank correlation coefficient is shown for each correlation. Only significant correlations are shown (P value < .05).

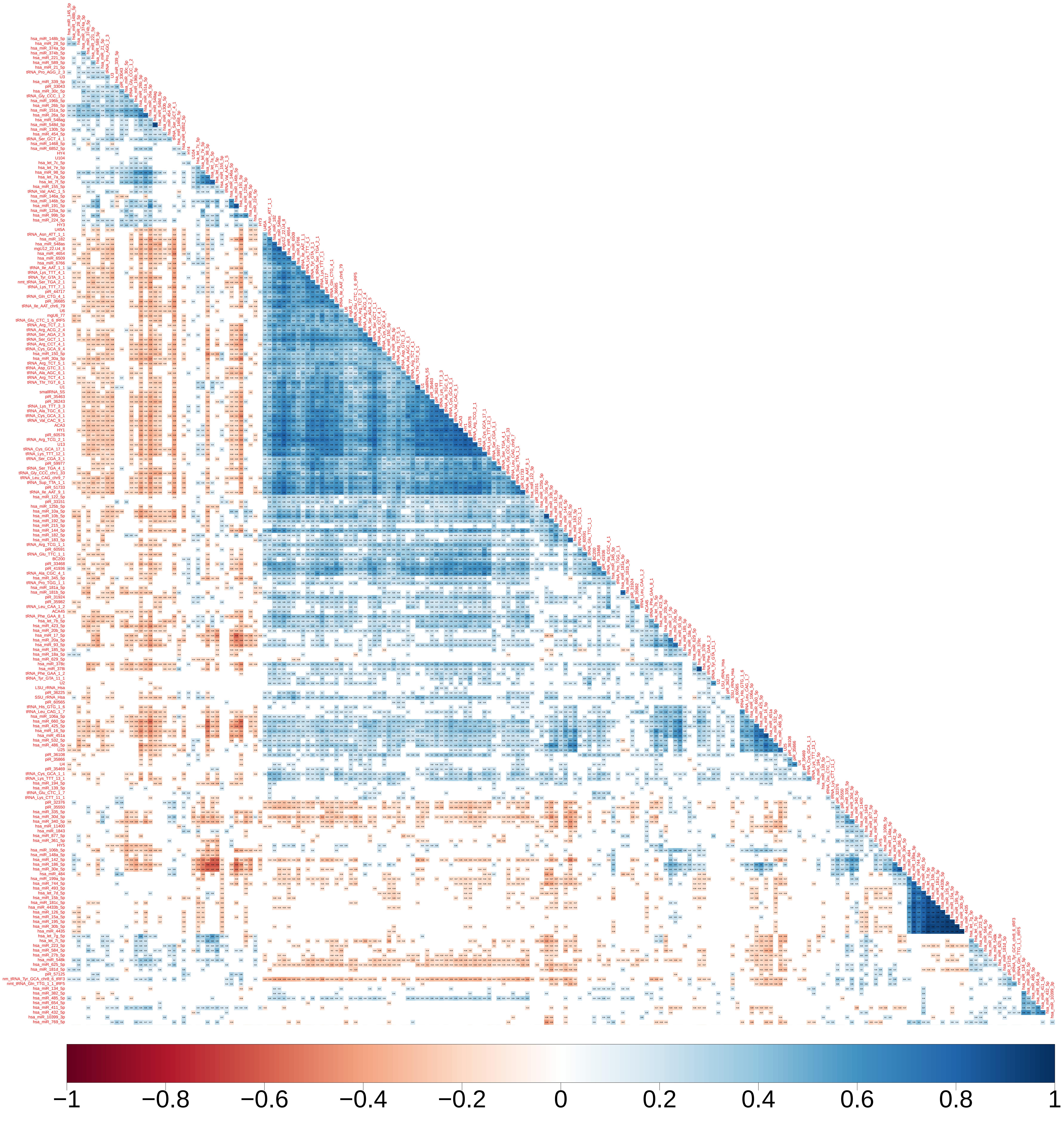
−1
−0.8
−0.6
−0.4
−0.2
0
0.2
0.4
0.6
0.8
1

eFigure 3 | A literature search was carried out using the MEDLINE-PubMed database from inception through 10 April 2023. For the database searches, terms related to “miRNA” or “gene” were combined with terms related to Alzheimer’s disease, cognition and brain. In each run, the miRNA’s and the gene name were changed.

((mir-224-5p) OR (SKIL)) AND ((alzheimer) OR (MCI) OR (mild cognitive impairment) OR (brain) OR (neuron) OR (neurodegeneration) OR (cognitive decline) OR (memory) OR (aging))

eTable 1 | Characteristics of the A+(T|N)+ study participants according to their A, T and N profile.

Variable	A+T+N+ participants	A+T+N- and A+T-N+ participants	P value
n	65	31	
Age (years)	77.3 (72.4, 80.2)	74.9 (69.8, 79.9)	0.343
Women [N (%)]	36 (55.4)	11 (35.5)	0.423
Body mass index (kg/m^2)	25.4 (23.4, 28.7)	25.5 (23.7, 28.4)	0.641
APOE ε4 carriers [N (%)]	43 (66.2)	16 (51.6)	0.171
Education (years)	8 (6, 10)	8 (6, 12)	0.849
Smoking [N (%)]			
Never	48 (73.8)	20 (64.5)	0.053
Former	12 (18.5)	10 (32.3)	0.024*
Current	5 (7.7)	1 (3.2)	0.770
Medication [N (%)]			
Antidepressant and anxiolytic	24 (36.9)	9 (29.0)	0.447
Antihypertensive	26 (40.0)	14 (45.2)	0.632
Statins	16 (24.6)	12 (38.7)	0.155
Other lipid-lowering drugs	4 (6.2)	2 (6.5)	0.978
MMSE at baseline (score)	25 (24, 27)	26 (24, 28)	0.402
A [N (%)]	65 (100.0)	31 (100.0)	1.000
T [N (%)]	65 (100.0)	28 (90.3)	0.011*
N [N (%)]	65 (100.0)	3 (9.7)	<0.001***
Follow-up (years)	1.7 (1.0, 2.7)	1.7 (1.0, 2.9)	0.896
Conversion to AD [N (%)]	47 (72.3)	22 (71.0)	0.891

Abbreviations: MMSE, Mini-mental state examination; AD, Alzheimer's disease.

Continuous data are presented as median (interquartile range), and categorical variables are presented as number (%). The Mann-Whitney test was used for comparison of non-normally distributed continuous variables, and the X^2 was used for comparison of categorical variables. Only three participants were A+T-N+ and consequently were joined with the A+T+N- group. *P value < .05, **P value < .01, ***P value < .001.

eTable 2 | Upregulated and downregulated Differentially Expressed sncRNAs comparing A+(T|N)+ with A-T-N-.

sncRNA	baseMean	log_2FC	lfcSE	stat	P value	Padj	Regulation
hsa-miR-130b-5p	5.420	0.330	0.137	2.402	0.016	0.565	Up
hsa-miR-146b-5p	1886.588	-0.194	0.087	-2.241	0.025	0.674	Down
hsa-miR-151a-5p	204.766	0.259	0.075	3.447	0.001	0.118	Up
hsa-miR-221-5p	6.494	0.389	0.129	3.014	0.003	0.134	Up
hsa-miR-26a-5p	10699.047	0.103	0.047	2.174	0.030	0.674	Up
hsa-miR-26b-5p	1836.808	0.088	0.043	2.062	0.039	0.674	Up
hsa-miR-30c-5p	96.306	0.167	0.082	2.033	0.042	0.674	Up
hsa-miR-339-5p	5.368	0.480	0.164	2.917	0.004	0.147	Up
hsa-miR-382-5p	6.025	-0.597	0.197	-3.035	0.002	0.134	Down
hsa-miR-548k	15.239	0.269	0.086	3.127	0.002	0.134	Up
hsa-miR-744-5p	88.619	0.167	0.080	2.099	0.036	0.674	Up
tRNA-Tyr-GTA-11-1	21.284	0.862	0.424	2.032	0.042	0.674	Up
U45A	108.560	-0.447	0.214	-2.089	0.037	0.674	Down

Padj using FDR. Padj < .05 was considered significant.

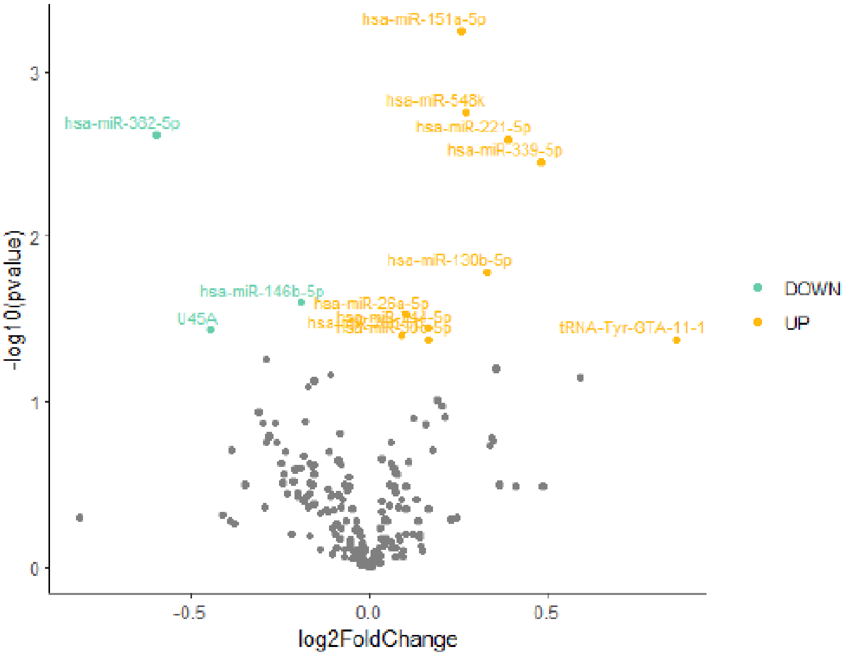

(A)

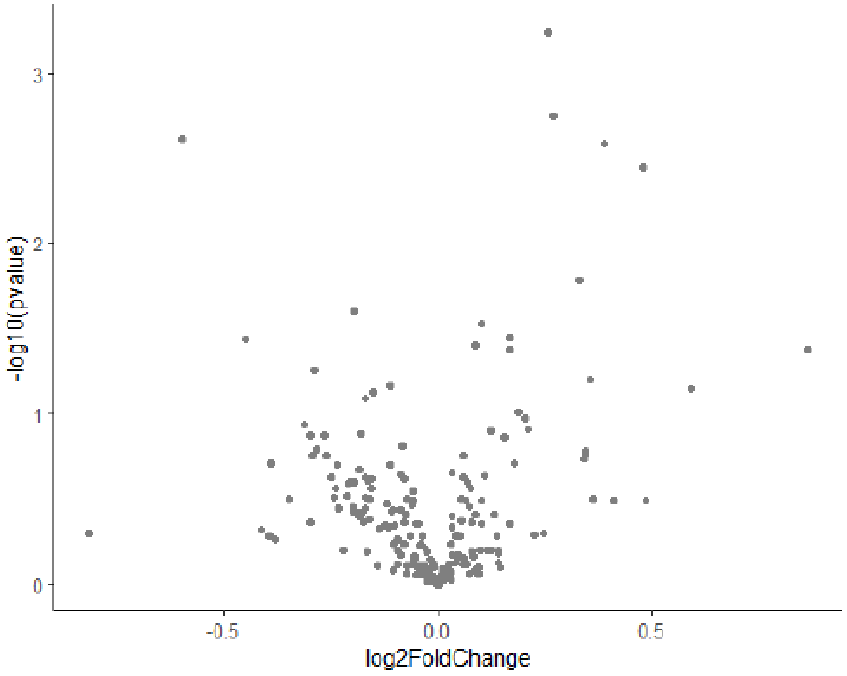

(B)

eFigure 4 | Volcano plot of upregulated and downregulated Differentially Expressed sncRNAs comparing A+(T|N)+ with A-T-N-. (A) sncRNAs with P value < .05, (B) sncRNAs with Padj < .05 after FDR.

eTable 3 | Conditional logistic regression analysis examining the individual associations between sncRNAs expression and A+(T|N)+.

	Unadjusted model			Model 1		
**sncRNA**	**OR (95% CI)**	**P value**	**Padj**	**OR (95% CI)**	**P value**	**Padj**
hsa-miR-130b-5p	1.389 (1.0334, 1.8669)	0.029	0.705	1.3243 (0.8367, 2.096)	0.231	0.994
hsa-miR-146b-5p	0.7328 (0.5452, 0.9851)	0.040	0.705	0.73 (0.4741, 1.1239)	0.153	0.994
hsa-miR-151a-5p	1.7397 (1.251, 2.4193)	0.001	0.208	1.6678 (0.9703, 2.8666)	0.064	0.994
hsa-miR-221-5p	1.5214 (1.1103, 2.0848)	0.009	0.312	1.1617 (0.7714, 1.7496)	0.473	0.994
hsa-miR-26a-5p	1.3664 (1.0166, 1.8367)	0.039	0.705	1.2099 (0.7566, 1.935)	0.426	0.994
hsa-miR-27b-5p	1.3263 (0.9915, 1.7742)	0.057	0.792	1.7286 (1.0805, 2.7653)	0.022	0.994
hsa-miR-30c-5p	1.3559 (1.0007, 1.8372)	0.049	0.781	1.3398 (0.7999, 2.2441)	0.266	0.994
hsa-miR-339-5p	1.6316 (1.1752, 2.2653)	0.003	0.236	1.7428 (1.0193, 2.98)	0.042	0.994
hsa-miR-382-5p	0.6456 (0.4729, 0.8814)	0.006	0.304	0.7356 (0.4657, 1.162)	0.188	0.994
hsa-miR-548ag	1.3584 (1.0131, 1.8215)	0.041	0.705	1.6379 (1.0379, 2.5846)	0.034	0.994
hsa-miR-548k	1.6149 (1.1846, 2.2014)	0.002	0.236	1.3953 (0.8811, 2.2097)	0.156	0.994
piR-31924	0.8858 (0.6534, 1.201)	0.435	0.984	0.6519 (0.4366, 0.9735)	0.037	0.994
piR-33043	1.3754 (1.0181, 1.858)	0.038	0.705	1.1593 (0.7557, 1.7784)	0.499	0.994
tRNA-Pro-AGG-2-3	1.5231 (1.1198, 2.0715)	0.007	0.304	1.3558 (0.8687, 2.1162)	0.180	0.994
LSU-rRNA-Hsa	1.0853 (0.8101, 1.4541)	0.583	0.984	0.6615 (0.4547, 0.9623)	0.031	0.994
U3	1.3709 (1.0234, 1.8364)	0.034	0.705	1.0957 (0.7138, 1.6819)	0.676	0.994
U4	1.2717 (0.9455, 1.7103)	0.112	0.984	1.631 (1.0302, 2.5822)	0.037	0.994

Abbreviations: OR, odds ratio; CI, confidence interval; MMSE, Mini-mental state examination.

Model 1 was adjusted for age, BMI, APOE ε4, smoking habit, education, use of anxiolytic or antidepressants, antihypertensive drugs, statins, and other lipid-lowering medication, and baseline MMSE. *Padj < .05, **Padj < .01, ***Padj < .001.

eTable 4 | Sensitivity analysis using logistic regression analysis to examine the individual associations between sncRNAs expression and A+(T|N)+.

	Unadjusted model			Model 1		
**sncRNA**	**OR (95% CI)**	**P value**	**Padj**	**OR (95% CI)**	**P value**	**Padj**
hsa-miR-130b-5p	1.392 (1.040, 1.888)	0.029	0.614	1.329 (0.842, 2.136)	0.227	0.990
hsa-miR-146b-5p	0.728 (0.537, 0.973)	0.035	0.614	0.726 (0.463, 1.114)	0.148	0.990
hsa-miR-151a-5p	1.713 (1.259, 2.390)	0.001	0.159	1.686 (0.999, 3.013)	0.061	0.990
hsa-miR-221-5p	1.530 (1.128, 2.131)	0.009	0.295	1.165 (0.768, 1.776)	0.469	0.990
hsa-miR-26a-5p	1.376 (1.030, 1.862)	0.034	0.614	1.215 (0.760, 1.977)	0.420	0.990
hsa-miR-26b-5p	1.364 (1.021, 1.843)	0.038	0.614	1.048 (0.649, 1.706)	0.850	0.990
hsa-miR-27b-5p	1.326 (0.994, 1.785)	0.058	0.806	1.742 (1.104, 2.870)	0.022	0.990
hsa-miR-30c-5p	1.354 (1.013, 1.836)	0.044	0.660	1.352 (0.810, 2.315)	0.257	0.990
hsa-miR-339-5p	1.675 (1.220, 2.371)	0.002	0.159	1.775 (1.060, 3.185)	0.040	0.990
hsa-miR-382-5p	0.638 (0.460, 0.830)	0.005	0.250	0.731 (0.451, 1.143)	0.182	0.990
hsa-miR-548ag	1.366 (1.022, 1.846)	0.038	0.614	1.651 (1.059, 2.676)	0.032	0.990
hsa-miR-548k	1.616 (1.197, 2.225)	0.002	0.159	1.401 (0.887, 2.256)	0.153	0.990
piR-31924	0.881 (0.619, 1.179)	0.415	0.994	0.647 (0.423, 0.992)	0.035	0.990
piR-33043	1.402 (1.045, 1.918)	0.028	0.614	1.167 (0.759, 1.830)	0.487	0.990
tRNA-Pro-AGG-2-3	1.525 (1.133, 2.095)	0.007	0.283	1.362 (0.868, 2.145)	0.177	0.990
LSU-rRNA-Hsa	1.082 (0.809, 1.502)	0.598	0.994	0.657 (0.441, 0.980)	0.029	0.990
U3	1.390 (1.041, 1.876)	0.028	0.614	1.100 (0.714, 1.706)	0.665	0.990
U4	1.264 (0.946, 1.721)	0.122	0.994	1.640 (1.053, 2.686)	0.036	0.990

Abbreviations: OR, odds ratio; CI, confidence interval; MMSE, Mini-mental state examination.

Model 1 was adjusted for age, sex, BMI, APOE ε4, smoking habit, education, use of anxiolytic or antidepressants, antihypertensive drugs, statins, and other lipid-lowering medication, and baseline MMSE. *Padj < .05, **Padj < .01, ***Padj < .001.

eTable 5 | Sensitivity analysis of sncRNAs associated with A+(T|N)+ in a logistic regression analysis. SncRNA are ranked from the highest to the lowest elastic net positive and negative regression coefficients for A+(T|N)+.

sncRNA	#a	β (95% CI)	sncRNA	#a	β (95% CI)
hsa-miR-339-5p	100	0.145 (0.136, 0.153)	hsa-miR-382-5p	100	-0.179 (-0.188, -0.170)
hsa-miR-548k	100	0.143 (0.131, 0.154)	hsa-miR-146b-5p	100	-0.131 (-0.141, -0.122)

Abbreviations: #a, Occurrence of metabolites (out of 100) in the elastic net conditional logistic regression; sncRNA, small non-coding RNA; hsa, Homo sapiens; CI, confidence interval.

eTable 6 | Upregulated and downregulated Differentially Expressed sncRNAs between AD-converters and AD non-converters.

sncRNA	baseMean	log_2FC	lfcSE	stat	P value	Padj	Regulation
hsa-let-7a-5p	3023.850	0.134	0.055	2.422	0.015	0.239	Up
hsa-let-7f-5p	12233.306	0.227	0.085	2.677	0.007	0.239	Up
hsa-miR-10a-5p	2344.771	-0.183	0.089	-2.056	0.040	0.331	Down
hsa-miR-130b-5p	5.420	0.316	0.141	2.250	0.024	0.255	Up
hsa-miR-134-5p	33.236	-0.331	0.149	-2.221	0.026	0.261	Down
hsa-miR-150-5p	62.127	-0.324	0.143	-2.266	0.023	0.255	Down
hsa-miR-151a-5p	204.766	0.293	0.077	3.801	0.000	0.030*	Up
hsa-miR-21-5p	6795.002	0.070	0.029	2.381	0.017	0.239	Up
hsa-miR-221-5p	6.494	0.410	0.131	3.136	0.002	0.089	Up
hsa-miR-26a-5p	10699.047	0.119	0.049	2.434	0.015	0.239	Up
hsa-miR-26b-5p	1836.808	0.119	0.044	2.712	0.007	0.239	Up
hsa-miR-30c-5p	96.306	0.209	0.084	2.474	0.013	0.239	Up
hsa-miR-345-5p	29.522	-0.186	0.090	-2.077	0.038	0.327	Down
hsa-miR-382-5p	6.025	-0.690	0.204	-3.376	0.001	0.051	Down
hsa-miR-485-5p	4.928	-0.445	0.214	-2.082	0.037	0.327	Down
hsa-miR-493-5p	56.626	-0.364	0.155	-2.357	0.018	0.239	Down
hsa-miR-548ag	7.793	0.249	0.117	2.136	0.033	0.309	Up
hsa-miR-548d-5p	9.170	0.273	0.107	2.551	0.011	0.239	Up
hsa-miR-548k	15.239	0.310	0.088	3.541	0.000	0.042*	Up
hsa-miR-98-5p	496.171	0.110	0.055	1.985	0.047	0.372	Up
piR-33043	51.833	0.478	0.197	2.427	0.015	0.239	Up
piR-35982	36.664	-0.530	0.202	-2.621	0.009	0.239	Down
tRNA-Arg-CCT-4-1	23.134	-0.534	0.222	-2.401	0.016	0.239	Down
tRNA-Cys-GCA-17-1	31.848	-0.373	0.189	-1.974	0.048	0.372	Down
SSU-rRNA-Hsa	42.313	-0.496	0.215	-2.311	0.021	0.241	Down
ACA45	40.174	-0.248	0.097	-2.563	0.010	0.239	Down
mgU6-77	11.710	-0.710	0.308	-2.310	0.021	0.241	Down

NOTE. Padj using FDR. Padj < 0.05 was considered significant.

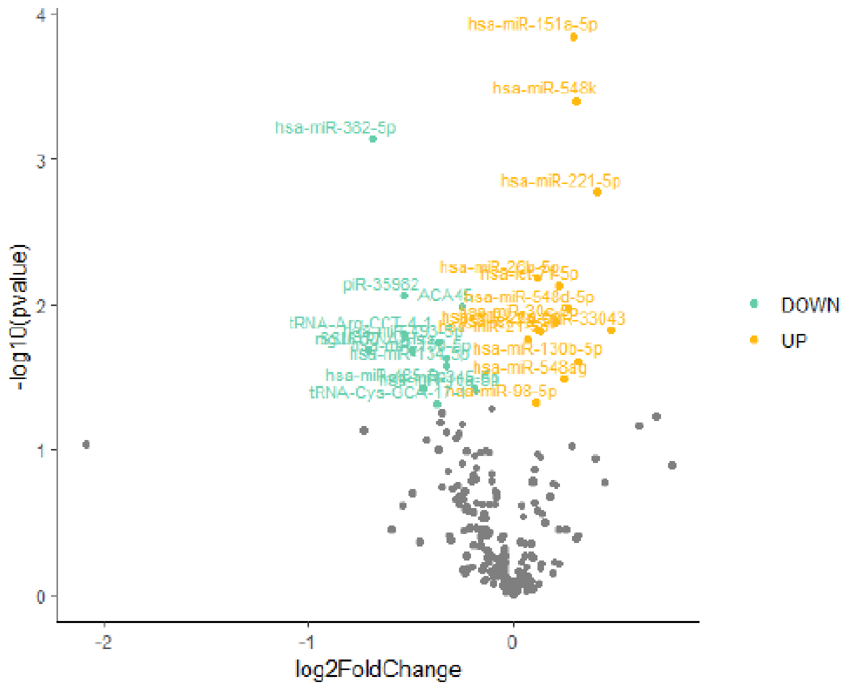

(A)

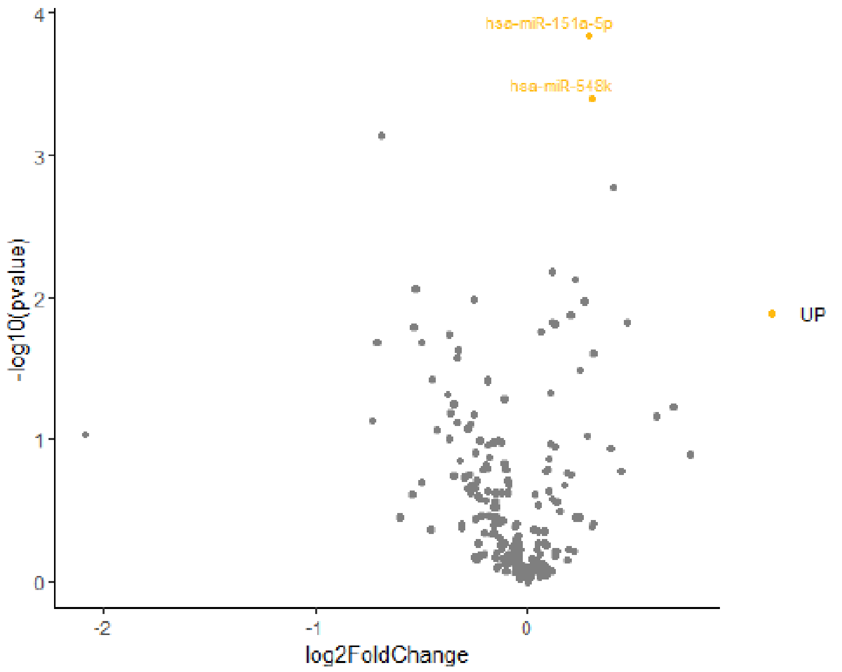

(B)

eFigure 5 | Volcano plot of upregulated and downregulated Differentially Expressed sncRNAs comparing AD-converters and AD non-converters. **(A)** sncRNAs with P value < .05, **(B)** sncRNAs with Padj < .05 after FDR.

eTable 7 | Cox regression analysis examining the individual associations between sncRNAs expression and risk of progression from MCI to AD.

	Unadjusted model			Model 1		
**sncRNA**	**HR (95% CI)**	**P value**	**Padj**	**HR (95% CI)**	**P value**	**Padj**
hsa-let-7a-5p	1.286 (1.030, 1.605)	0.026	0.361	1.157 (0.911, 1.470)	0.233	0.964
hsa-let-7f-5p	1.348 (1.056, 1.721)	0.017	0.270	1.306 (1.036, 1.646)	0.024	0.677
hsa-miR-106b-5p	0.880 (0.691, 1.122)	0.303	0.853	0.670 (0.454, 0.987)	0.043	0.677
hsa-miR-146b-5p	0.946 (0.744, 1.203)	0.653	0.899	1.378 (1.047, 1.814)	0.022	0.677
hsa-miR-15a-5p	0.659 (0.295, 1.471)	0.308	0.853	0.206 (0.066, 0.639)	0.006	0.645
hsa-miR-151a-5p	1.508 (1.214, 1.872)	<0.001	0.042*	1.086 (0.860, 1.371)	0.491	0.964
hsa-miR-181c-5p	0.821 (0.542, 1.245)	0.354	0.853	0.449 (0.211, 0.958)	0.038	0.677
hsa-miR-191-5p	0.969 (0.766, 1.227)	0.795	0.949	1.370 (1.036, 1.813)	0.028	0.677
hsa-miR-20a-5p	0.893 (0.701, 1.136)	0.357	0.853	0.707 (0.510, 0.978)	0.037	0.677
hsa-miR-21-5p	1.288 (1.027, 1.616)	0.029	0.361	1.178 (0.861, 1.611)	0.307	0.964
hsa-miR-221-5p	1.310 (1.084, 1.581)	0.005	0.152	1.179 (0.918, 1.513)	0.197	0.964
hsa-miR-26a-5p	1.293 (1.026, 1.631)	0.030	0.361	1.051 (0.823, 1.34)	0.692	0.999
hsa-miR-26b-5p	1.348 (1.055, 1.722)	0.017	0.270	1.127 (0.863, 1.471)	0.382	0.964
hsa-miR-30c-5p	1.321 (1.060, 1.647)	0.013	0.270	1.043 (0.837, 1.298)	0.709	0.999
hsa-miR-30e-5p	1.064 (0.847, 1.335)	0.596	0.899	0.742 (0.551, 0.999)	0.049	0.677
hsa-miR-339-5p	1.357 (1.109, 1.661)	0.003	0.152	1.004 (0.795, 1.269)	0.970	0.999
hsa-miR-382-5p	0.670 (0.508, 0.884)	0.005	0.152	0.803 (0.598, 1.078)	0.144	0.841
hsa-miR-493-5p	0.582 (0.352, 0.961)	0.034	0.396	0.772 (0.481, 1.238)	0.282	0.964
hsa-miR-548ag	1.315 (1.048, 1.648)	0.018	0.270	1.199 (0.933, 1.54)	0.156	0.841
hsa-miR-548d-5p	1.351 (1.070, 1.705)	0.011	0.261	1.311 (1.012, 1.700)	0.041	0.677
hsa-miR-548k	1.514 (1.194, 1.921)	0.001	0.062	1.215 (0.927, 1.593)	0.158	0.841
hsa-miR-584-5p	1.207 (0.960, 1.516)	0.107	0.853	1.851 (1.375, 2.491)	<0.001	<0.001***

hsa-miR-99b-5p	1.159 (0.918, 1.463)	0.216	0.853	1.321 (1.012, 1.724)	0.041	0.677
piR-33043	1.398 (1.132, 1.72)	0.002	0.125	1.258 (0.995, 1.592)	0.056	0.722
piR-33151	1.330 (1.090, 1.624)	0.005	0.152	1.164 (0.907, 1.494)	0.234	0.964
piR-44717	1.129 (0.922, 1.384)	0.241	0.853	1.223 (1.008, 1.487)	0.042	0.677
tRNA-Asp-GTC-3-1	1.141 (0.916, 1.421)	0.238	0.853	1.275 (1.009, 1.611)	0.042	0.677
tRNA-Glu-CTC-1-7	0.985 (0.794, 1.221)	0.889	0.974	1.339 (1.005, 1.783)	0.046	0.677
tRNA-Gly-CCC-1-2	1.331 (1.077, 1.645)	0.008	0.213	1.113 (0.878, 1.412)	0.377	0.964
tRNA-Pro-AGG-2-3	1.261 (1.040, 1.528)	0.018	0.270	0.970 (0.736, 1.278)	0.827	0.999
tRNA-Ser-GCT-4-1	1.130 (0.909, 1.404)	0.271	0.853	1.364 (1.019, 1.825)	0.037	0.677

Abbreviations: HR, hazard ratio; CI, confidence interval; MMSE, Mini-mental state examination.

Model 1 was adjusted for ATN at baseline, age, sex, BMI, *APOE* ε4, smoking habit, education, use of anxiolytic or antidepressants, antihypertensive drugs, statins, and other lipid-lowering medication, and baseline MMSE. *Padj < .05, **Padj < .01, ***Padj < .001.

eTable 8 | Genes regulated by the ATN-related sncRNA signature.

Gene Symbol	Gene Name	miRNA regulation	Functions related to AD	References
ILF3	Interleukin enhancer binding factor 3	↑ hsa-miR-221-5p<br>↓ hsa-miR-382-5p	Transcriptional suppressor of the LOAD risk gene *HLA-DQA1*, which has been found upregulated in AD patient's hippocampus.	(4,5)
CTNNB1	Catenin Beta 1	↑ hsa-miR-221-5p	When the Wnt/β-catenin (*CTNNB1*) is activated, β-catenin facilitates gene transcription, while when this pathway is inactivated, β-catenin is degraded. Mutations of *CTNNB1* disturb synaptic plasticity, neuronal apoptosis, and neurogenesis.	(6,7)
FAM98B	Family with sequence similarity 98 member B	↓ hsa-miR-146b-5p<br>↓ hsa-miR-382-5p	It shuttles between the nucleus and the cytoplasm, transporting RNAs, and modulates the translation of specific neuronal mRNAs.	(1,2)
DDX6	DEAD-box helicase 6	↑ hsa-miR-548k<br>↑ hsa-miR-144-5p	Tau binds directly to DDX6 and increases miRNAs miR-21, miR-124 and let-7 silencing activity, which have been related to AD pathology.	(8)
CARHSP1	Calcium regulated heat stable protein 1	↑ hsa-miR-548k<br>↑ hsa-miR-144-5p	Oligodendrogenic transcription factor downregulated in the brain of Ulk4$^{tm1a/tm1a}$ mice, characterized by impaired myelination.	(3)
DDX21	DExD-box helicase 21	↓ hsa-miR-146b-5p<br>↑ hsa-miR-339-5p	RNA helicase which coordinates ribosomal RNA transcription and processing, RNA editing, RNA transport, and nuclear and mitochondrial splicing.<br>Downregulated in the anterior cingulate of bipolar disorder patients.	(4)

RNF19A	Ring finger protein 19A	↑ hsa-miR-221-5p<br>↑ hsa-miR-339-5p	Rnf19a-deficient mice have been found to have reduced adult neurogenesis and enhanced long-term potentiation in the dentate gyrus. Also implicated in amyotrophic lateral sclerosis and Parkinson's disease.	(9)
SKIL	SKI like proto-oncogene	↑ hsa-miR-548k<br>↓ hsa-miR-382-5p	Promotes the expression of cell proliferation genes and concomitantly represses the differentiation genes in granule neuron precursors of mice cerebellum.<br>It also plays a dual role as a corepressor or coactivator of *TGFβ*-induced transcription in postmitotic neurons and drives axonal growth.	(10,11)
CBX6	Chromobox 6	↑ hsa-miR-221-5p<br>↓ hsa-miR-146b-5p	Causative gene associated with *Nbg15* for the development of T2D, a risk factor for AD.	(12)
HNRNPA1	Heterogeneous nuclear ribonucleoprotein A1	↑ hsa-miR-221-5p<br>↑ hsa-miR-339-5p<br>↓ hsa-mir-382-5p	Affects the alternative splicing of an exon of *CD33*. This gene is an immune receptor on the membrane of microglia and macrophages, have been found increased in human AD brains and correlated with plaque burden as well as insoluble $A\beta_{42}$ levels. Mice lacking Cd33 showed a reduced burden of amyloid plaques and insoluble $A\beta_{42}$. Regulation of *CD33* splicing through *HNRNPA1* is associated with reduced risk of AD and decreased suppressive signaling on microglial activity.	(13–15)
CUL3	Cullin 3	↑ hsa-miR-144-5p<br>↑ hsa-miR-339-5p	Downregulation of the ubiquitin ligase (E3) gene *CUL3* in brain exacerbates AD through *NRF2* aggregation and impaired oxidative stress protection.	(16,17)
SMAD4	SMAD family member 4	↑ hsa-miR-144-5p<br>↑ hsa-miR-221-5p<br>↑ hsa-miR-339-5p	Tgfb1(-/-) mice with *Smad4* inhibition exhibit highly increased neuronal apoptosis.<br>Mice with *Smad4* blockage exhibited *BACE* upregulation, *APP* degradation, tangle formation and Aβ generation in the brain.	(18,19)

MYLIP	Myosin regulatory light chain interacting protein	↑ hsa-miR-548k<br>↑ hsa-miR-221-5p<br>↑ hsa-miR-339-5p	Promotes the ubiquitylation and subsequent lysosomal degradation of LDLR. Genetic ablation of *MYLIP* increases LDLR, which facilitates Aβ uptake and clearance by microglia in the brains of APP/PS1 mice. Also, *MYLIP* inhibition upregulates lysosomal/phagocytic genes in microglia.	(7)
SOD2	Superoxide dismutase 2	↑ hsa-miR-144-5p<br>↑ hsa-miR-221-5p<br>↑ hsa-miR-339-5p<br>↓ hsa-miR-382-5p	Antioxidant enzyme that maintains the control of ROS. *SOD2* polymorphisms have been associated with increased susceptibility to oxidative stress. The relationship between *SOD2* rs4880 polymorphism and AD have yielded inconsistent results, but in combination with *APOε4* allele carriage it increases the risk for MCI and AD.	(20,21)
PRPF8	Pre-mRNA processing factor 8	↑ hsa-miR-144-5p<br>↑ hsa-miR-221-5p<br>↓ hsa-miR-146b-5p<br>↓ hsa-miR-382-5p	Regulation of glutamatergic neurotransmission in brain cortex. Homozygous mice expressing the aberrant Prpf8 variants developed atrophy pf the cerebellum because of extensive granule cell loss.	(22,23)

Abbreviations: LOAD, Late Onset Alzheimer’s disease; AD, Alzheimer’s disease; T2D, type 2 diabetes; ROS, reactive oxygen species; LDLR, low density lipoprotein receptor; MCI, mild cognitive impairment.

eTable 9 | Genes regulated by the AD-related sncRNA signature.

Gene Symbol	Gene Name	miRNA Regulation	Function related to AD	References
B2M	Beta-2-microglobulin	↑ hsa-miR-548d-5p<br>↓ hsa-miR-224-5p	*B2M* is part of the MHC I complex, which exhibits increased expression in samples from AD patients, and impairs neuronal plasticity, neurite growth, and neurite regeneration.<br>It is a membrane marker of senescence and blood biomarker of AD. The increase in *B2M* is related to a decline in cognitive scores, to the pathological changes of Aβ (low CSF Aβ1-42), but not to neurodegeneration or tau pathology.<br>Compared with young mice, old mice experience elevated *B2M* levels in their hippocampus and plasma.	(24–27)
GAPDH	Glyceraldehyde-3-phosphate dehydrogenase	↓ hsa-miR-224-5p<br>↑ hsa-miR-221-5p	Interaction between *GAPDH* and AD proteins through oxidative stress in brain. Specimens of AD, including Aβ, Aβ precursor protein and Tau, leads to impairment of the *GAPDH* glycolytic function in AD and thus in apoptosis. Proapoptotic factor abundant in the blood of AD patients.	(28–31)
SEC24C	SEC24 homolog C, COPII coat complex component	↑ hsa-miR-877-5p<br>↓ hsa-miR-224-5p<br>↑ hsa-miR-221-5p	The family of SEC24p oversee vesicle trafficking, including shaping vesicle, cargo selection and concentration, of subunits of the γ-secretase (involved in Aβ production). *SEC24C* transports the γ-subunit nicastrin.	(32)
ARIH2	Ariadne RBR E3 ubiquitin protein ligase 2	↓ hsa-miR-224-5p<br>↓ hsa-miR-382-5p	In spinal cord injury, *ARIH2* overexpression promotes neurotrophic growth factor expression, brain-derived neurotrophic factor expression, astrocyte and neuronal viability, neuroprotective cytokine pleiotrophin, neurite outgrowth, and suppressed astrocyte apoptosis. Knockdown of *ARIH2*-derived Triad1 protein has the opposite effects.	(33)

ELAVL1	ELAV like RNA binding protein 1	↓ hsa-miR-625-5p<br>↑ hsa-miR-221-5p	Downregulated *ELAVL1* may reduce the abundance and type of mRNA in AD patients through modulation of mRNA stability and promotion of variable cleavage of precursor RNA.	(34,35)
TNRC6A	Trinucleotide repeat containing adaptor 6A	↑ hsa-miR-548d-5p<br>↓ hsa-miR-769-5p<br>↓ hsa-miR-224-5p	*TNRC6A* promotes dendritic growth, both in the hippocampus and cerebellum, by regulating global somatodendritic translation and actin cytoskeletal dynamics of developing neurons. Perturbation of this protein results in reduced dendritic growth of cultured hippocampal neurons.	(36)
EPAS1	Endothelial PAS domain protein 1	↓ hsa-miR-625-5p<br>↓ hsa-miR-224-5p	This oxygen-dependent transcription factor is the major regulator of *EPO*, which has neuroprotective and pro-angiogenesis effects, and triggers responses to hypoxia including metabolic adaptation and angiogenesis.	(37)
TNRC6B	Trinucleotide repeat containing adaptor 6B	↓ hsa-miR-625-5p<br>↓ hsa-miR-224-5p<br>↑ hsa-miR-221-5p<br>↓ hsa-miR-454-5p	Inhibits translation. In ADHD, heterozygous pathogenic variant in *TNRC6B* have been found.	(38,39)
AGO2	Argonaute RISC catalytic component 2	↑ hsa-miR-548d-5p<br>↑ hsa-miR-548k<br>↓ hsa-miR-625-5p	It cleaves mRNA to regulate gene expression. Astrocytes exposed to $A\beta_{1-42}$ oligomers show loss of Ago2 phosphorylation, which leads to uncontrolled production of pro-inflammatory cytokines and neuroinflammation, leading to loss of neurons.<br>*BACE1* and *APP* protein levels are significantly elevated following *AGO2* silencing in cultured human brain cells.	(40,41)
ATXN1L	Ataxin 1 like	↑ hsa-miR-548k<br>↑ hsa-miR-877-5p	*ATXN1* may help to shuttle *ATXN1L* into the nucleus where they interact in a large protein complex. Therefore, it is related to Aβ pathology mediated by *BACE1* expression. Also related to progressive non-fluent aphasia.	(42)

CSNK1E	Casein kinase 1 epsilon	↑ hsa-miR-221-5p<br>↓ hsa-miR-382-5p	Involved in circadian rhythms during early AD development. The expression of Csnk1E was higher in AD mice compared to control mice after light induction.	(43)
ATXN1	Ataxin 1	↑ hsa-miR-877-5p<br>↓ hsa-miR-625-5p<br>↓ hsa-miR-224-5p<br>↓ hsa-miR-454-5p<br>↓ hsa-miR-382-5p	Knockout of Atxn1 increases *BACE1* levels and enhances amyloidogenic cleavage of APP, exacerbating Aβ deposition and gliosis in AD mouse models, and impaired hippocampal neurogenesis and olfactory axonal targeting.	(44)
GAB1	GRB2 associated binding protein 1	↑ hsa-miR-548d-5p<br>↑ hsa-miR-877-5p	Promotes hippocampal LTP. In AD patients and in a mouse model of AD it is decreased, inhibiting septal cholinergic neurons projecting to the hippocampus.	(45)
CRKL	CRK like proto-oncogene, adaptor protein	↑ hsa-miR-877-5p<br>↓ hsa-miR-769-5p	Some of the neurotoxic effects of Aβ peptides may be mediated via the activation of proteins belonging to the Abl family, that regulate actin cytoskeleton structure through *CRKL* and phosphorylate microtubule-associated Tau protein, thus weakening synaptic transmission.	(46)
NF2	Moesin-ezrin-radixin like (MERLIN) tumor suppressor	↓ hsa-miR-769-5p<br>↑ hsa-miR-221-5p	Loss of function of the gene lead to the development of schwannomas, meningiomas and juvenile cataracts.	(47)
AKT1	AKT serine/threonine kinase 1	↑ hsa-miR-548k<br>↓ hsa-miR-382-5p	The PI3K/Akt signaling pathway is anti-apoptotic, and *AKT1* inhibits Akt phosphorylation (activation), which in turn upregulates the activity of *GSK3β*, a kinase implicated in the pathogenesis of AD.<br>ROS-mediated oxidative modification of Akt1 leads to reduced synaptic Akt1-mTOR signaling which contributes to synaptic dysfunction in AD. It is rescued by overexpression of Akt1 in APP/PS1 mice.	(48,49)

HSP90AA1	Heat shock protein 90 alpha family class A member 1	↑ hsa-miR-548d-5p<br>↑ hsa-miR-548k<br>↓ hsa-miR-625-5p<br>↓ hsa-miR-224-5p	Regulates the stabilization and function of p-tau in AD. Inhibition of Hsp90 in AD mouse models reduced Aβ toxicity and normalizes synaptic function.<br>In another study, *HSP90AA1* was decreased in the blood from MCI and AD and was negatively correlated with α and β secretase activities.	(50,51)
RICTOR	RPTOR independent companion of MTOR complex 2	↓ hsa-miR-769-5p<br>↓ hsa-miR-625-5p	Regulates transcription of multiple genes involved in oxidative phosphorylation and other mitochondrial functions. Inhibits nuclear mitochondrial expression. It also controls the actin cytoskeleton polymerization.<br>Some studies find it not altered in AD. Other studies find that Rictor conditional knockout impairs long-term memory and late long-term potentiation, involved in spatial memory.	(52,53)
CTNNB1	Catenin Beta 1	↑ hsa-miR-548d-5p<br>↑ hsa-miR-548k	When the Wnt/β-catenin (*CTNNB1*) is activated, β-catenin facilitates gene transcription, while when this pathway is inactivated, β-catenin is degraded. Mutations of *CTNNB1* disturb synaptic plasticity, neuronal apoptosis, and neurogenesis.	(6,7)
OLA1	Obg like ATPase 1	↓ hsa-miR-769-5p<br>↓ hsa-miR-382-5p	Antioxidant suppressor. Downregulated *OLA1* preserves neuronal components.	(54)
AP3S2	Adaptor related protein complex 3 subunit sigma 2	↑ hsa-miR-548d-5p<br>↑ hsa-miR-548k<br>↓ hsa-miR-769-5p	Encodes a protein involved in protein transport whose variants are associated with the risk of T2D (which is a risk factor for AD).	(55)

YWHAG	Tyrosine 3-monooxygenase/tryptophan 5-monooxygenase activation protein gamma	↑ hsa-miR-548d-5p<br>↓ hsa-miR-224-5p<br>↓ hsa-miR-454-5p	Involved in signal transduction. Expression levels are increased in CSF specimens from AD patients and in the hippocampus of 5XFAD mice compared to controls. Recurrent distal deletion of *YWHAG* has been identified in patients with intellectual disability and neurobehavioral problems.	(56,57)
MAPK1	Mitogen-activated protein kinase 1	↓ hsa-miR-769-5p<br>↓ hsa-miR-454-5p<br>↓ hsa-miR-382-5p	The AKT-MAPK1-MTORC1 pathways is activated in 5XFAD mice (which has Aβ pathology). *MAPK1* phosphorylates the transcription factor EB, a master regulator of the autophagy-lysosomal pathway, thus inactivating it and promoting the development of AD.	(58)
NUDT21	Nudix hydrolase 21	↑ hsa-miR-548k<br>↓ hsa-miR-625-5p<br>↓ hsa-miR-454-5p	Elevated *NUDT21* results in an enrichment of inefficiently translated long mRNA isoforms, which is associated with intellectual disability and neuropsychiatric disease.<br>Contrary, other studies indicate that reduce *NUDT21* function, such as in *Nudt21*$^{+/-}$ mice, cause learning deficits, cortical hyperexcitability, and misregulated alternative polyadenylation in the hippocampi.	(59,60)
ARL6IP1	ADP ribosylation factor like GTPase 6 interacting protein 1	↑ hsa-miR-548d-5p<br>↑ hsa-miR-548k<br>↓ hsa-miR-224-5p<br>↓ hsa-miR-382-5p	Elevated in the brain of AD Tg2576 mice and AB_{1-42} treated brain CECs. Promotes apoptosis through interaction with *ASK1*.	(61)

Abbreviations: *EPO*, Erytrhopoietin; ADHD, Attention aeficit and hyperactivity disorder; LTP, Long-term potentiation; Abl, Abelson non-receptor tyrosine kinase; PNC, Peripheral nervous system; NSC, neural stem cells; CEC, Cerebral microvascular endothelial cells; *ASK1*, Apoptosis signal-regulating kinase 1.

eTable 10 | Significantly enriched KEGG pathways for the ATN-related sncRNA signature.

KEGG ID	KEGG term	Gene Set Size	Counts	Padj	Genes
hsa05200	MicroRNAs in cancer	150	44	<0.001	*ABCC1, ABL1, BAK1, BCL2L11, BCL2L2, BMI1, BRCA1, CASP3, CCND1, CCND2, CCNG1, CDCA5, CDK6, CDKN1A, COMMD3-BMI1, CRKL, CYP1B1, DICER1, FZD3, HDAC4, HOXD10, KRAS, MAP2K1, MDM4, MMP16, MTOR, NFKB1, NOTCH1, PDGFRA, PDGFRB, PLCG2, PRKCA, PTEN, RHOA, SHC4, SLC7A1, SOS1, SOS2, STAT3, TGFB2, TNC, TP63, ZEB1, ZEB2*
hsa04722	Neurotrophin signaling pathway	119	27	0.029	*ABL1, AKT3, ARHGDIA, CALM1, CALML4, CAMK4, CRKL, GAB1, IRAK1, IRAK4, KRAS, MAP2K1, NFKB1, NTF3, NTRK2, PDPK1, PLCG2, RAP1A, RAP1B, RAPGEF1, RHOA, SHC4, SORT1, SOS1, SOS2, TRAF6, YWHAE, NOTCH1, PDGFRA, PDGFRB, PLCG2, PRKCA, PTEN, RHOA, SHC4, SLC7A1, SOS1, SOS2, STAT3, TGFB2, TNC, TP63, ZEB1, ZEB2*
hsa04550	Signaling pathways regulating pluripotency of stem cells	139	31	0.021	*ACVR1B, AKT3, BMI1, BMPR1A, COMMD3-BMI1, DLX5, DVL1, FZD1, FZD10, FZD2, FZD3, FZD5, FZD7, ID4, IGF1, IL6ST, JARID2, KAT6A, KRAS, MAP2K1, MEIS1, NEUROG1, PCGF5, SKIL, SMAD1, SMAD3, SMAD5, STAT3, WNT16, WNT5A, WNT9B, PRKCA, PTEN, RHOA,*

					SHC4, SLC7A1, SOS1, SOS2, STAT3, TGFB2, TNC, TP63, ZEB1, ZEB2
hsa04390	Hippo signaling pathway	154	34	0.019	*ACTG1, BMP2, BMP8B, BMPR1A, CCND1, CCND2, CSNK1D, CTNNA3, DVL1, FZD1, FZD10, FZD2, FZD3, FZD5, FZD7, LIMD1, MPP5, NF2, PARD6B, PRKCZ, SMAD1, SMAD3, TEAD1, TGFB2, TGFBR1, WNT16, WNT5A, WNT9B, WWTR1, YAP1, YWHAB, YWHAE, YWHAQ, YWHAZ, SHC4, SLC7A1, SOS1, SOS2, STAT3, TGFB2, TNC, TP63, ZEB1, ZEB2*
hsa04120	Ubiquitin mediated proteolysis	136	29	0.041	*BRCA1, CBL, CBLB, CUL3, CUL4A, HERC2, KLHL13, KLHL9, NEDD4L, PIAS2, RHOBTB1, SKP1, SMURF2, TRAF6, UBE2A, UBE2B, UBE2E1, UBE2E3, UBE2G1, UBE2H, UBE2J1, UBE2K, UBE2QL1, UBE3A, UBE4A, UBR5, VHL, WWP1, XIAP, YAP1, YWHAB, YWHAE, YWHAQ, YWHAZ, SHC4, SLC7A1, SOS1, SOS2, STAT3, TGFB2, TNC, TP63, ZEB1, ZEB2*
hsa05205	Proteoglycans in cancer	198	42	0.014	*ACTG1, AKT3, CASP3, CBL, CCND1, CDKN1A, CTTN, DDX5, ERBB4, FZD1, FZD10, FZD2, FZD3, FZD5, FZD7, GAB1, GPC1, HOXD10, IGF1, ITGAV, ITGB1, ITPR1, KRAS, MAP2K1, MSN, MTOR, PDPK1, PLCG2, PRKCA, RHOA, RRAS2, SDC2, SDC4, SOS1, SOS2, STAT3, TGFB2, TIAM1, TLR4, WNT16, WNT5A, WNT9B, ZEB1, ZEB2*

hsa05202	Transcriptional misregulation in cancer	186	39	0.019	*BAK1, BCL11B, BCL6, BMI1, CCND2, CCNT1, CDK14, CDKN1A, COMMD3-BMI1, DDX5, ELK4, EWSR1, HDAC2, HIST1H3F, HIST1H3G, IGF1, IL6, KLF3, KMT2A, MEF2C, MEIS1, MLLT1, MYCN, NCOR1, NFKB1, NSD2, PAX5, POLK, RUNX1T1, RUNX2, RXRA, SIN3A, SMAD1, SP1, SPI1, SS18, TLX3, WNT16, ZEB1, WNT16, WNT5A, WNT9B, ZEB1, ZEB2*
hsa05414 1	Protein processing in endoplasmic reticulum	165	34	0.037	*ATXN3, BAG2, BAK1, CKAP4, DAD1, DDOST, DNAJC10, DNAJC3, EDEM3, EIF2AK3, ERLEC1, ERO1A, HERPUD1, HSP90AA1, HSPA1B, HYOU1, MARCH6, NFE2L2, NSFL1C, P4HB, SAR1A, SAR1B, SEC62, SEC63, SKP1, SSR2, STT3A, TUSC3, UBE2G1, UBE2J1, UBQLN2, UGGT1, XBP1, YOD1, SPI1, SS18, TLX3, WNT16, ZEB1, WNT16, WNT5A, WNT9B, ZEB1, ZEB2*
hsa04010	MAPK signaling pathway	295	55	0.025	*AKT3, ANGPT4, ATF2, CACNA2D3, CASP3, CRKL, DUSP7, EFNA5, ELK4, ERBB4, FGF7, GNG12, HSPA1B, IGF1, IL1B, IL1RAP, IRAK1, IRAK4, KIT, KITLG, KRAS, MAP2K1, MAP3K2, MAP4K3, MAP4K4, MAPK8IP1, MAPKAPK5, MECOM, MEF2C, NFATC1, NFATC3, NFKB1, NTF3, NTRK2, PDGFRA, PDGFRB, PLA2G4A, PPM1A, PPP3CA, PPP3CB, PRKCA, PTPRR, RAP1A, RAP1B*
hsa05200	Pathways in cancer	524	97	0.001	*ABL1, ADCY2, AKT3, APPL1, AR, BAK1, BCL2L11, BMP2, CALM1, CALML4, CASP3, CBL, CCDC6, CCND1, CCND2,*

	CDK6, CDKN1A, COL4A1, CRKL, CTBP2, CTNNA3, DLL1, DVL1, F2R, F2RL3, FGF7, FZD1, FZD10, FZD2, FZD3, FZD5, FZD7, GNA13, GNG12, GSTA1, GSTM2, HDAC2, HHIP, HSP90AA1, IFNA21, IFNA4, IGF1, IL23A, IL6

eTable 11 | Significantly enriched KEGG pathways for the AD-related sncRNA signature.

KEGG ID	KEGG term	Gene Set Size	Counts	Padj	Genes
hsa05206	MicroRNA in cancer	150	67	<0.001	*ABCC1, ABL1, ATM, BAK1, BCL2, BCL2L2, BMF, BMI1, BMPR2, BRCA1, CASP3, CCND1, CCND2, CCNG1, CD44, CDC25C, CDCA5, CDK6, CDKN1B, COMMD3-BMI1, CREBBP, CRKL, CYP1B1, DDIT4, DICER1, DNMT3B, EFNA3, ERBB2, FZD3, HDAC4, HNRNPK, HOXD10, IGF2BP1, IRS1, KRAS, MAP2K1, MAP2K2, MAPK1, MARCKS, MCL1, MDM4, MMP16, MTOR, NFKB1, NOTCH1, NOTCH2, PDCD4, PDGFB, PDGFRA, PDGFRB, PLCG2, PRKCA, PTEN, RPS6KA5, SHC4, SIRT1, SLC7A1, SOS1, SOS2, STAT3, TGFB2, TNC, TP63, TPM1, TRIM71, ZEB1, ZEB2*
hsa03015	mRNA surveillance pathway	91	38	<0.001	*ACIN1, BCL2L2-PABPN1, CASC3, CPSF2, CPSF6, CPSF7, CSTF2, CSTF2T, ETF1, GSPT1, GSPT2, MSI2, NCBP2, NUDT21, NXF1, PABPC1, PABPC1L2A, PABPC1L2B, PABPC4, PABPC5, PABPN1, PAPOLA, PAPOLG, PNN, PPP1CA, PPP2CA, PPP2R1A, PPP2R2C, PPP2R2D, RBM8A, SAP18, SMG1, SMG5, SRRM1, UPF2, UPF3B, WDR33, WDR82*
hsa04550	Signalling pathways regulating pluripotency of stem cells	139	58	<0.001	*ACVR1B, ACVR2A, ACVR2B, AKT2, AKT3, AXIN2, BMI1, BMPR2, COMMD3-BMI1, DLX5, DUSP9, DVL3, FZD10, FZD2, FZD3, FZD5, FZD6, FZD7, GSK3B, HAND1, IGF1, IGF1R, IL6ST, INHBA, INHBC, ISL1, JAK3, JARID2, KAT6A, KLF4, KRAS, LEFTY2, MAP2K1, MAP2K2, MAPK1, MEIS1, NEUROG1, OTX1, PAX6, PCGF3, PCGF5, PIK3CB, PIK3R3, REST, RIF1, SKIL, SMAD2, SMAD3, SMAD4, SMAD5, SMAD9, SOX2, STAT3, TBX3, WNT16, WNT5A, WNT7B, WNT9B*

hsa04390	Hippo signaling pathway	154	57	<0.001	*ACTB, ACTG1, AMOT, AXIN2, BBC3, BMP8B, BMPR2, CCND1, CCND2, CDH1, CSNK1D, CTNNA3, DVL3, FZD10, FZD2, FZD3, FZD5, FZD6, FZD7, GDF6, GSK3B, LATS1, LATS2, LEF1, LIMD1, MOB1A, MOB1B, NKD1, PARD3, PARD6B, PPP1CA, PPP2CA, PPP2R1A, PPP2R2C, PPP2R2D, PRKCI, PRKCZ, SERPINE1, SMAD2, SMAD3, SMAD4, SOX2, TCF7L2, TEAD1, TGFB2, TGFBR1, TP73, WNT16, WNT5A, WNT7B, WNT9B, WWTR1, YAP1, YWHAE, YWHAG, YWHAQ, YWHAZ*
hsa04218	Cellular senescence	160	59	<0.001	*AKT2, AKT3, ATM, CACNA1D, CALM1, CALML3, CALML4, CAPN2, CCNB1, CCND1, CCND2, CDK1, CDK4, CDK6, CHEK2, ETS1, FOXM1, HIPK1, HIPK2, HIPK3, HLA-A, HLA-G, ITPR1, KRAS, MAP2K1, MAP2K2, MAPK1, MTOR, NBN, NFATC1, NFATC3, NFKB1, PIK3CB, PIK3R3, PPP1CA, PPP3CA, PPP3CB, PPP3R1, PTEN, RAD1, RASSF5, RBL2, RELA, RRAS2, SERPINE1, SIRT1, SLC25A5, SMAD2, SMAD3, SQSTM1, TGFB2, TGFBR1, TRAF3IP2, TRPM7, TSC1, VDAC1, VDAC2, ZFP36L1, ZFP36L2*
hsa04141	Protein processing in endoplasmic reticulum	165	56	0.002	*ATXN3, BAK1, BCAP31, BCL2, CALR, CAPN2, DAD1, DDIT3, DDOST, DERL1, DNAJC10, DNAJC3, EDEM3, EIF2AK2, EIF2AK3, ERO1A, GANAB, HERPUD1, HSP90AA1, HSPA8, HSPH1, HYOU1, MAN1A1, MAN1A2, MAPK8, MAPK9, MARCH6, NFE2L2, NSFL1C, P4HB, SAR1A, SAR1B, SEC23A, SEC24A, SEC24C, SEC62, SEC63, SEL1L, SKP1, SSR1, SSR3, STT3A, SVIP, SYVN1, TRAM1, TUSC3, UBE2D1, UBE2D2, UBE2D3, UBE2G1, UBE2J1, UBQLN2, UBQLN4, UGGT1, XBP1, YOD1*
hsa04120	Ubiquitin mediated proteolysis	136	46	<0.011	*ANAPC1, BRCA1, CBL, CBLB, CDC27, CUL4A, CUL5, DDB1, FBXW8, HERC2, HERC4, HUWE1, KLHL13, KLHL9, NEDD4L, PIAS1, PIAS2, PIAS3, RHOBTB1, SIAH1, SKP1, SMURF2, SYVN1, TRIM37, UBA6, UBE2A, UBE2B, UBE2D1, UBE2D2, UBE2D3, UBE2E1, UBE2F, UBE2G1, UBE2H, UBE2J1, UBE2K, UBE2N, UBE2QL1, UBE2R2, UBE2Z, UBE4A, UBOX5, UBR5, VHL, WWP1, XIAP*

hsa05202	Transcriptional misregulation in cancer	186	62	<0.001	*AFF1, ATM, BAK1, BCL11B, BMI1, CCND2, CCNT1, CCNT2, CD40, CDKN1B, COMMD3-BMI1, DDIT3, DDX5, ELK4, ETV1, ETV6, EWSR1, EYA1, FEV, FLI1, H3F3B, HDAC2, HIST1H3B, HIST1H3F, HIST1H3G, HOXA10, HPGD, IGF1, IGF1R, KLF3, KMT2A, MAF, MEF2C, MEIS1, MEN1, MITF, MLLT1, MLLT3, MYCN, NCOR1, NFKB1, PAX5, PAX7, PLAT, POLK, PPARG, PRCC, PROM1, RELA, RUNX1, RUNX2, RXRA, SIN3A, SIX4, SP1, SS18, SUPT3H, TFE3, TLX3, TRAF1, WNT16, ZEB1*
hsa04010	MAPK signaling pathway	295	91	<0.001	*AKT2, AKT3, ANGPT2, ANGPT4, ARRB1, CACNA1D, CACNA1E, CACNA2D3, CACNB1, CACNB4, CACNG7, CASP3, CDC42, CHUK, CRKL, DDIT3, DUSP10, DUSP7, DUSP8, DUSP9, EFNA3, EFNA5, ELK1, ELK4, ERBB2, ERBB4, EREG, FAS, FGF7, GNG12, HSPA8, IGF1, IGF1R, IGF2, IL1B, IRAK4, JUND, KIT, KITLG, KRAS, MAP2K1, MAP2K2, MAP2K4, MAP3K2, MAP4K3, MAP4K4, MAPK1, MAPK8, MAPK8IP1, MAPK9, MAPKAPK5, MAPT, MECOM, MEF2C, MKNK2, NF1, NFATC1, NFATC3, NFKB1, NLK, NTF3, NTRK2, PAK2, PDGFB, PDGFRA, PDGFRB, PPM1A, PPP3CA, PPP3CB, PPP3R1, PPP5C, PRKACA, PRKCA, PTPRR, RAC1, RAP1A, RAP1B, RASA2, RASGRF1, RASGRP1, RELA, RPS6KA4, RPS6KA5, RPS6KA6, RRAS2, SOS1, SOS2, TAOK1, TAOK2, TGFB2, TGFBR1*

hsa05200	Pathways in cancer	524	153	<0.001	*ABL1, ADCY2, ADCY3, AGTR1, AKT2, AKT3, APPL1, AR, AXIN2, BAK1, BBC3, BCL2, CALM1, CALML3, CALML4, CAMK2A, CASP3, CASP7, CBL, CCND1, CCND2, CDC42, CDH1, CDK4, CDK6, CDKN1B, CHUK, COL4A1, COL4A3, CREBBP, CRKL, CSF2RB, CTNNA3, CXCR4, DVL3, EDNRA, EGLN1, ELK1, EPO, ERBB2, ESR1, ETS1, F2RL3, FAS, FGF7, FZD10, FZD2, FZD3, FZD5, FZD6, FZD7, GNA13, GNAI1, GNAI2, GNAQ, GNB4, GNG11, GNG12, GNG7, GSK3B, GSTA1, GSTM2, GSTM3, HDAC2, HEY1, HEYL, HSP90AA1, IFNA21, IFNGR2, IGF1, IGF1R, IGF2, IL12RB2, IL13RA1, IL23A, IL5, IL6R, IL6ST, IL7R, ITGA6, ITGAV, ITGB1, JAK3, KIT, KITLG, KRAS, LAMC1, LEF1, LPAR1, MAP2K1, MAP2K2, MAPK1, MAPK8, MAPK9, MECOM, MITF, MTOR, NCOA3, NFE2L2, NFKB1, NOS2, NOTCH1, NOTCH2, NQO1, PDGFB, PDGFRA, PDGFRB, PIK3CB, PIK3R3, PLCB1, PLCB4, PLCG2, POLK, PPARG, PRKACA, PRKCA, PTEN, PTGER3, RAC1, RAD51, RALA, RALBP1, RASGRP1, RASSF5, RELA, RPS6KA5, RPS6KB1, RUNX1, RXRA, SKP1, SMAD2, SMAD3, SMAD4, SOS1, SOS2, SP1, STAT1, STAT2, STAT3, SUFU, TCF7L2, TGFB2, TGFBR1, TPM3, TPR, TRAF1, TRAF4, VHL, WNT16, WNT5A, WNT7B, WNT9B, XIAP*

eTable 12 | Significantly enriched GO terms in the ATN-related sncRNA signature.

GO ID	GO Term	Gene Set Size	Counts	Padj
**Biological process**				
GO: 0022008	Neurogenesis	1551	264	<0.001
GO: 0033554	Cellular response to stress	1867	311	<0.001
GO: 0010629	Negative regulation of gene expression	1733	285	<0.001
GO: 0070727	Cellular macromolecule localization	1825	296	<0.001
GO: 0045935	Positive regulation of nucleobase-containing compound metabolic process	1847	298	<0.001
GO: 0009719	Response to endogenous stimulus	1595	253	<0.001
GO: 0007267	Cell-cell signaling	1575	249	<0.001
GO: 0051049	Regulation of transport	1765	276	<0.001
GO: 0006928	Movement of cell or subcellular component	1967	299	<0.001
GO: 0009888	Tissue development	1925	288	<0.001
**Molecular function**				
GO: 0019904	Protein domain specific binding	684	142	<0.001
GO: 0019787	Ubiquitin-like protein transferase activity	416	79	<0.001
GO: 0019900	Kinase binding	711	124	<0.001
GO: 0008134	Transcription factor binding	638	111	<0.001
GO: 0003700	DNA-binding transcription factor activity	1700	269	<0.001
GO: 0051020	GTPase binding	646	102	0.008
GO: 0003723	RNA binding	1603	248	<0.001
GO: 0008092	Cytoskeletal protein binding	940	140	0.009
GO: 0046914	Transition metal ion binding	1058	153	0.016
GO: 0017076	Purine nucleotide binding	1865	261	0.002
**Cellular component**				
GO: 0030054	Cell junction	1268	220	<0.001
GO: 0044451	Nucleoplasm part	1087	178	<0.001
GO: 0005694	Chromosome	1014	162	<0.001
GO: 0097458	Neuron part	1690	269	<0.001
GO: 0005730	Nucleolus	939	144	<0.001
GO: 1902494	Catalytic complex	1346	202	<0.001
GO: 0005794	Golgi apparatus	1516	226	<0.001
GO: 0098805	Whole membrane	1630	229	<0.001
GO: 0005783	Endoplasmic reticulum	1861	250	<0.001
GO: 0005887	Integral component of plasma membrane	1596	213	<0.001

Abbreviations: GO, Gene ontology.

eTable 13 | Significantly enriched GO terms in the AD-related sncRNA signature.

GO ID	GO Term	Gene Set Size	Counts	Padj
**Biological process**				
GO: 0010629	Negative regulation of gene expression	1733	546	<0.001
GO: 0022008	Neurogenesis	1551	462	<0.001
GO: 0070647	Protein modification by small protein conjugation or removal	1028	303	<0.001
GO: 0009719	Response to endogenous stimulus	1595	470	<0.001
GO: 0070727	Cellular macromolecule localization	1825	533	<0.001
GO: 2000026	Regulation of multicellular organismal development	1908	552	<0.001
GO: 0009891	Positive regulation of biosynthetic process	1949	557	<0.001
GO: 0046907	Intracellular transport	1803	508	<0.001
GO: 0033554	Cellular response to stress	1867	520	<0.001
GO: 0008283	Cell proliferation	1986	537	<0.001
**Molecular function**				
GO: 0019787	Ubiquitin-like protein transferase activity	416	139	<0.001
GO: 0019904	Protein domain specific binding	684	217	<0.001
GO: 0019901	Protein kinase binding	631	187	<0.001
GO: 0003700	DNA-binding transcription factor activity	1700	500	<0.001
GO: 0003723	RNA binding	1603	449	<0.001
GO: 0051020	GTPase binding	646	169	<0.001
GO: 0032553	Ribonucleotide binding	1865	466	<0.001
GO: 0046914	Transition metal ion binding	1058	264	<0.001
GO: 0042802	Identical protein binding	1696	413	<0.001
GO: 0005102	Signalling receptor binding	1538	363	<0.001
**Cellular component**				
GO: 0044451	Nucleoplasm part	1087	329	<0.001
GO: 0097458	Neuron part	1690	479	<0.001
GO: 0005694	Chromosome	1014	278	<0.001
GO: 0005730	Nucleolus	939	255	<0.001
GO: 1902494	Catalytic complex	1346	365	<0.001
GO: 0005794	Golgi apparatus	1516	403	<0.001
GO: 0098805	Whole membrane	1630	416	<0.001
GO: 0005783	Endoplasmic reticulum	1861	454	<0.001
GO: 0031226	Intrinsic component of plasma membrane	1673	381	<0.001
GO: 0044430	Cytoskeletal part	1620	364	<0.001

Abbreviations: GO, Gene Ontology.

REFERENCES

1. EMBL-EBII. Ensembl (https://www.ensembl.org/Homo_sapiens/Info/Index). 2023.
2. MiRBase. miRBase (https://www.mirbase.org/ftp.shtml). 2019.
3. Wang Lab PNGCU of P. DASHR (https://dashr2.lisanwanglab.org/download.php).
4. Zhang X, Zou M, Wu Y, Jiang D, Wu T, Zhao Y, et al. Regulation of the Late Onset alzheimer’s Disease Associated HLA-DQA1/DRB1 Expression. Am J Alzheimers Dis Other Demen. 2022 Jan 26;37:153331752210850.
5. Dharshini SAP, Taguchi Y h., Gromiha MM. Investigating the energy crisis in Alzheimer disease using transcriptome study. Sci Rep. 2019 Dec 6;9(1):18509.
6. Keshavarzi M, Moradbeygi F, Mobini K, Ghaffarian Bahraman A, Mohammadi P, Ghaedi A, et al. The interplay of aryl hydrocarbon receptor/WNT/CTNNB1/Notch signaling pathways regulate amyloid beta precursor mRNA/protein expression and effected the learning and memory of mice. Toxicol Res (Camb). 2022 Feb 26;11(1):147–61.
7. Zhuang W, Ye T, Wang W, Song W, Tan T. CTNNB1 in neurodevelopmental disorders. Front Psychiatry. 2023 Mar 16;14.
8. Chauderlier A, Gilles M, Spolcova A, Caillierez R, Chwastyniak M, Kress M, et al. Tau/DDX6 interaction increases microRNA activity. Biochimica et Biophysica Acta (BBA) - Gene Regulatory Mechanisms. 2018 Aug;1861(8):762–72.
9. Park H, Yang J, Kim R, Li Y, Lee Y, Lee C, et al. Mice lacking the PSD-95–interacting E3 ligase, Dorfin/Rnf19a, display reduced adult neurogenesis, enhanced long-term potentiation and impaired contextual fear conditioning. Sci Rep. 2015 Nov 10;5(1):16410.
10. Chen X, Chanda A, Ikeuchi Y, Zhang X, Goodman J V., Reddy NC, et al. The Transcriptional Regulator SnoN Promotes the Proliferation of Cerebellar Granule Neuron Precursors in the Postnatal Mouse Brain. The Journal of Neuroscience. 2019 Jan 2;39(1):44–62.
11. Bonni S, Bonni A. SnoN signaling in proliferating cells and postmitotic neurons. FEBS Lett. 2012 Jul 4;586(14):1977–83.
12. Altenhofen D, Khuong JMA, Kuhn T, Lebek S, Görigk S, Kaiser K, et al. E96V Mutation in the Kdelr3 Gene Is Associated with Type 2 Diabetes Susceptibility in Obese NZO Mice. Int J Mol Sci. 2023 Jan 3;24(1):845.

13. Griciuc A, Patel S, Federico AN, Choi SH, Innes BJ, Oram MK, et al. TREM2 Acts Downstream of CD33 in Modulating Microglial Pathology in Alzheimer's Disease. Neuron. 2019 Sep;103(5):820-835.e7.

14. Griciuc A, Serrano-Pozo A, Parrado AR, Lesinski AN, Asselin CN, Mullin K, et al. Alzheimer's Disease Risk Gene CD33 Inhibits Microglial Uptake of Amyloid Beta. Neuron. 2013 May;78(4):631–43.

15. Komuro R, Honda Y, Yanaizu M, Nagahama M, Kino Y. Alzheimer's Disease-Associated Alternative Splicing of CD33 Is Regulated by the HNRNPA Family Proteins. Cells. 2023 Feb 13;12(4):602.

16. Wang Q, Li WX, Dai SX, Guo YC, Han FF, Zheng JJ, et al. Meta-Analysis of Parkinson's Disease and Alzheimer's Disease Revealed Commonly Impaired Pathways and Dysregulation of NRF2-Dependent Genes. Journal of Alzheimer's Disease. 2017 Feb 20;56(4):1525–39.

17. Liu D, Dai SX, He K, Li GH, Liu J, Liu LG, et al. Identification of hub ubiquitin ligase genes affecting Alzheimer's disease by analyzing transcriptome data from multiple brain regions. Sci Prog. 2021 Jan 23;104(1):003685042110011.

18. Brionne TC, Tesseur I, Masliah E, Wyss-Coray T. Loss of TGF-β1 Leads to Increased Neuronal Cell Death and Microgliosis in Mouse Brain. Neuron. 2003 Dec;40(6):1133–45.

19. Chou PY, Lin SR, Lee MH, Schultz L, Sze CI, Chang NS. A p53/TIAF1/WWOX triad exerts cancer suppression but may cause brain protein aggregation due to p53/WWOX functional antagonism. Cell Communication and Signaling. 2019 Dec 17;17(1):76.

20. Gamarra D, Elcoroaristizabal X, Fernández-Martínez M, de Pancorbo MM. Association of the C47T Polymorphism in SOD2 with Amnestic Mild Cognitive Impairment and Alzheimer's Disease in Carriers of the APOEε4 Allele. Dis Markers. 2015;2015:1–7.

21. Peculis R, Konrade I, Skapare E, Fridmanis D, Nikitina-Zake L, Lejnieks A, et al. Identification of glyoxalase 1 polymorphisms associated with enzyme activity. Gene. 2013 Feb;515(1):140–3.

22. Cáceres A, González JR. When pitch adds to volume: coregulation of transcript diversity predicts gene function. BMC Genomics. 2018 Dec 13;19(1):926.

23. Krausová M, Kreplová M, Banik P, Cvačková Z, Kubovčiak J, Modrák M, et al. Retinitis pigmentosa–associated mutations in mouse Prpf8 cause misexpression of circRNAs and degeneration of cerebellar granule cells. Life Sci Alliance. 2023 Jun 5;6(6):e202201855.

24. Lau SF, Cao H, Fu AKY, Ip NY. Single-nucleus transcriptome analysis reveals dysregulation of angiogenic endothelial cells and neuroprotective glia in Alzheimer's disease. Proceedings of the National Academy of Sciences. 2020 Oct 13;117(41):25800–9.

25. Lee H, Brott BK, Kirkby LA, Adelson JD, Cheng S, Feller MB, et al. Synapse elimination and learning rules co-regulated by MHC class I H2-Db. Nature. 2014 May 8;509(7499):195–200.

26. Huang YM, Ma YH, Gao PY, Wang ZB, Huang LY, Hou JH, et al. Plasma β2-microglobulin and cerebrospinal fluid biomarkers of Alzheimer's disease pathology in cognitively intact older adults: the CABLE study. Alzheimers Res Ther. 2023 Apr 1;15(1):69.

27. Smith LK, He Y, Park JS, Bieri G, Snethlage CE, Lin K, et al. β2-microglobulin is a systemic pro-aging factor that impairs cognitive function and neurogenesis. Nat Med. 2015 Aug 6;21(8):932–7.

28. Butterfield DA, Hardas SS, Lange MLB. Oxidatively Modified Glyceraldehyde-3-Phosphate Dehydrogenase (GAPDH) and Alzheimer's Disease: Many Pathways to Neurodegeneration. Journal of Alzheimer's Disease. 2010 Apr 1;20(2):369–93.

29. Tsai CW, Tsai CF, Lin KH, Chen WJ, Lin MS, Hsieh CC, et al. An investigation of the correlation between the S-glutathionylated GAPDH levels in blood and Alzheimer's disease progression. PLoS One. 2020 May 29;15(5):e0233289.

30. El Kadmiri N, Slassi I, El Moutawakil B, Nadifi S, Tadevosyan A, Hachem A, et al. Glyceraldehyde-3-phosphate dehydrogenase (GAPDH) and Alzheimer's disease. Pathologie Biologie. 2014 Dec;62(6):333–6.

31. Sunaga K, Takahashi H, Chuang DM, Ishitani R. Glyceraldehyde-3-phosphate dehydrogenase is over-expressed during apoptotic death of neuronal cultures and is recognized by a monoclonal antibody against amyloid plaques from Alzheimer's brain. Neurosci Lett. 1995 Nov;200(2):133–6.

32. Wouters R, Michiels C, Sannerud R, Kleizen B, Dillen K, Vermeire W, et al. Assembly of γ-secretase occurs through stable dimers after exit from the endoplasmic reticulum. Journal of Cell Biology. 2021 Sep 6;220(9).

33. Wu C, Xu G, Bao G, Gao H, Chen J, Zhang J, et al. Ubiquitin ligase Triad1 promotes neurite outgrowth by inhibiting MDM2-mediated ubiquitination of the neuroprotective factor pleiotrophin. Journal of Biological Chemistry. 2022 Oct;298(10):102443.

34. Li J, Chen F, Zhang Q, Meng X, Yao X, Risacher SL, et al. Genome-wide Network-assisted Association and Enrichment Study of Amyloid Imaging Phenotype in Alzheimer's Disease. Curr Alzheimer Res. 2020 Jan 10;16(13):1163–74.

35. Zhang X, Zou M, Wu Y, Jiang D, Wu T, Zhao Y, et al. Regulation of the Late Onset alzheimer's Disease Associated *HLA-DQA1/DRB1* Expression. Am J Alzheimers Dis Other Demen. 2022 Jan 26;37:153331752210850.

36. Nawalpuri B, Sharma A, Chattarji S, Muddashetty RS. Distinct temporal expression of the GW182 paralog TNRC6A in neurons regulates dendritic arborization. J Cell Sci. 2021 Aug 15;134(16).

37. Cristante E, Liyanage SE, Sampson RD, Kalargyrou A, De Rossi G, Rizzi M, et al. Late neuroprogenitors contribute to normal retinal vascular development in a *Hif2a* -dependent manner. Development. 2018 Jan 1;

38. Eising E, Carrion-Castillo A, Vino A, Strand EA, Jakielski KJ, Scerri TS, et al. A set of regulatory genes co-expressed in embryonic human brain is implicated in disrupted speech development. Mol Psychiatry. 2019 Jul 20;24(7):1065–78.

39. Granadillo JL, P.A. Stegmann A, Guo H, Xia K, Angle B, Bontempo K, et al. Pathogenic variants in *TNRC6B* cause a genetic disorder characterised by developmental delay/intellectual disability and a spectrum of neurobehavioural phenotypes including autism and ADHD. J Med Genet. 2020 Oct;57(10):717–24.

40. Vilardo E, Barbato C, Ciotti M, Cogoni C, Ruberti F. MicroRNA-101 Regulates Amyloid Precursor Protein Expression in Hippocampal Neurons. Journal of Biological Chemistry. 2010 Jun;285(24):18344–51.

41. De D, Mukherjee I, Guha S, Paidi RK, Chakrabarti S, Biswas SC, et al. Rheb-mTOR activation rescues Aβ-induced cognitive impairment and memory function by restoring miR-146 activity in glial cells. Mol Ther Nucleic Acids. 2021 Jun;24:868–87.

42. Carlson KM, Melcher L, Lai S, Zoghbi HY, Clark HB, Orr HT. Characterization of the Zebrafish *atxn1/axh* Gene Family. J Neurogenet. 2009 Jan;23(3):313–23.

43. Bellanti F, Iannelli G, Blonda M, Tamborra R, Villani R, Romano A, et al. Alterations of Clock Gene RNA Expression in Brain Regions of a Triple Transgenic Model of Alzheimer's Disease. Journal of Alzheimer's Disease. 2017 Jul 17;59(2):615–31.

44. Suh J, Romano DM, Nitschke L, Herrick SP, DiMarzio BA, Dzhala V, et al. Loss of Ataxin-1 Potentiates Alzheimer's Pathogenesis by Elevating Cerebral BACE1 Transcription. Cell. 2019 Aug;178(5):1159-1175.e17.

45. Lu NN, Tan C, Sun NH, Shao LX, Liu XX, Gao YP, et al. Cholinergic Grb2-Associated-Binding Protein 1 Regulates Cognitive Function. Cerebral Cortex. 2018 Jul 1;28(7):2391–404.

46. Reichenstein M, Borovok N, Sheinin A, Brider T, Michaelevski I. Abelson Kinases Mediate the Depression of Spontaneous Synaptic Activity Induced by Amyloid Beta 1–42 Peptides. Cell Mol Neurobiol. 2021 Apr 12;41(3):431–48.

47. Claudio JO, Lutchman M, Rouleau GA. Widespread but cell type-specific expression of the mouse neurofibromatosis type 2 gene. Neuroreport. 1995 Oct;6(14):1942–6.

48. Ahmad F, Singh K, Das D, Gowaikar R, Shaw E, Ramachandran A, et al. Reactive Oxygen Species-Mediated Loss of Synaptic Akt1 Signaling Leads to Deficient Activity-Dependent Protein Translation Early in Alzheimer's Disease. Antioxid Redox Signal. 2017 Dec;27(16):1269–80.

49. Zu G, Sun K, Li L, Zu X, Han T, Huang H. Mechanism of quercetin therapeutic targets for Alzheimer disease and type 2 diabetes mellitus. Sci Rep. 2021 Nov 25;11(1):22959.

50. Sulistio YA, Heese K. The Ubiquitin-Proteasome System and Molecular Chaperone Deregulation in Alzheimer's Disease. Mol Neurobiol. 2016 Mar 7;53(2):905–31.

51. Qian X hang, Liu X li, Chen S di, Tang H dong. Integrating peripheral blood and brain transcriptomics to identify immunological features associated with Alzheimer's disease in mild cognitive impairment patients. Front Immunol. 2022 Sep 9;13.

52. Sun YX, Ji X, Mao X, Xie L, Jia J, Galvan V, et al. Differential Activation of mTOR Complex 1 Signaling in Human Brain with Mild to Severe Alzheimer's Disease. Journal of Alzheimer's Disease. 2013 Nov 13;38(2):437–44.

53. Lian B, Liu M, Lan Z, Sun T, Meng Z, Chang Q, et al. Hippocampal overexpression of SGK1 ameliorates spatial memory, rescues Aβ pathology and actin cytoskeleton polymerization in middle-aged APP/PS1 mice. Behavioural Brain Research. 2020 Apr;383:112503.

54. Croze E, Yamaguchi KD, Knappertz V, Reder AT, Salamon H. Interferon-beta-1b-induced short- and long-term signatures of treatment activity in multiple sclerosis. Pharmacogenomics J. 2013 Oct 19;13(5):443–51.

55. Xiu X, Zhang H, Xue A, Cooper DN, Yan L, Yang Y, et al. Genetic evidence for a causal relationship between type 2 diabetes and peripheral artery disease in both Europeans and East Asians. BMC Med. 2022 Aug 31;20(1):300.

56. Park H, Lee YB, Chang KA. miR-200c suppression increases tau hyperphosphorylation by targeting 14-3-3γ in early stage of 5xFAD mouse model of Alzheimer's disease. Int J Biol Sci. 2022;18(5):2220–34.

57. Sathe G, Na CH, Renuse S, Madugundu AK, Albert M, Moghekar A, et al. Quantitative Proteomic Profiling of Cerebrospinal Fluid to Identify Candidate Biomarkers for Alzheimer's Disease. Proteomics Clin Appl. 2019 Jul 25;13(4):1800105.

58. Zheng X, Lin W, Jiang Y, Lu K, Wei W, Huo Q, et al. Electroacupuncture ameliorates beta-amyloid pathology and cognitive impairment in Alzheimer disease via a novel mechanism involving activation of TFEB (transcription factor EB). Autophagy. 2021 Nov 2;17(11):3833–47.

59. Gennarino VA, Alcott CE, Chen CA, Chaudhury A, Gillentine MA, Rosenfeld JA, et al. NUDT21-spanning CNVs lead to neuropsychiatric disease and altered MeCP2 abundance via alternative polyadenylation. Elife. 2015 Aug 27;4.

60. Alcott CE, Yalamanchili HK, Ji P, van der Heijden ME, Saltzman A, Elrod N, et al. Partial loss of CFIm25 causes learning deficits and aberrant neuronal alternative polyadenylation. Elife. 2020 Apr 22;9.

61. Wang H, Fan L, Wang H, Ma X, Du Z. Amyloid β Regulates the Expression and Function of AIP1. Journal of Molecular Neuroscience. 2015 Jan 2;55(1):227–32.
